# Solution-Processed Thin Film Transparent Photovoltaics: Present Challenges and Future Development

**DOI:** 10.1007/s40820-024-01547-6

**Published:** 2024-10-23

**Authors:** Tianle Liu, Munerah M. S. Almutairi, Jie Ma, Aisling Stewart, Zhaohui Xing, Mengxia Liu, Bo Hou, Yuljae Cho

**Affiliations:** 1 UM-SJTU Joint Institute, Shanghai Jiao Tong University, Shanghai, 200240 People’s Republic of China; 2 School of Physics and Astronomy, Cardiff University, Cardiff, CF24 3AA Wales, UK; 3 Department of Electrical and Computer Engineering, Yale University, New Haven, CT 06511 USA; 4 Energy Sciences Institute, Yale University, West Haven, CT 06516 USA; 5 Future Photovoltaics Research Center, Global Institute of Future Technology, Shanghai Jiao Tong University, Shanghai, 200240 People’s Republic of China

**Keywords:** Transparent semiconductors, Solution-processable transparent solar cell, Emerging solar cell materials, Building-integrated photovoltaics

## Abstract

Electrical energy is essential for modern society to sustain economic growths. The soaring demand for the electrical energy, together with an awareness of the environmental impact of fossil fuels, has been driving a shift towards the utilization of solar energy. However, traditional solar energy solutions often require extensive spaces for a panel installation, limiting their practicality in a dense urban environment. To overcome the spatial constraint, researchers have developed transparent photovoltaics (TPV), enabling windows and facades in vehicles and buildings to generate electric energy. Current TPV advancements are focused on improving both transparency and power output to rival commercially available silicon solar panels. In this review, we first briefly introduce wavelength- and non-wavelength-selective strategies to achieve transparency. Figures of merit and theoretical limits of TPVs are discussed to comprehensively understand the status of current TPV technology. Then we highlight recent progress in different types of TPVs, with a particular focus on solution-processed thin-film photovoltaics (PVs), including colloidal quantum dot PVs, metal halide perovskite PVs and organic PVs. The applications of TPVs are also reviewed, with emphasis on agrivoltaics, smart windows and facades. Finally, current challenges and future opportunities in TPV research are pointed out.

## Highlights

- Recent advancement in solution-processed thin film transparent photovoltaics (TPVs) is summarized, including perovskites, organics, and colloidal quantum dots.
- Pros and cons of the emerging TPVs are analyzed according to the materials characteristics and the application requirements on the aesthetics and energy generation.
- Promising TPV applications are discussed with emphasis on agrivoltaics, smart windows and facades.

## Graphical abstract

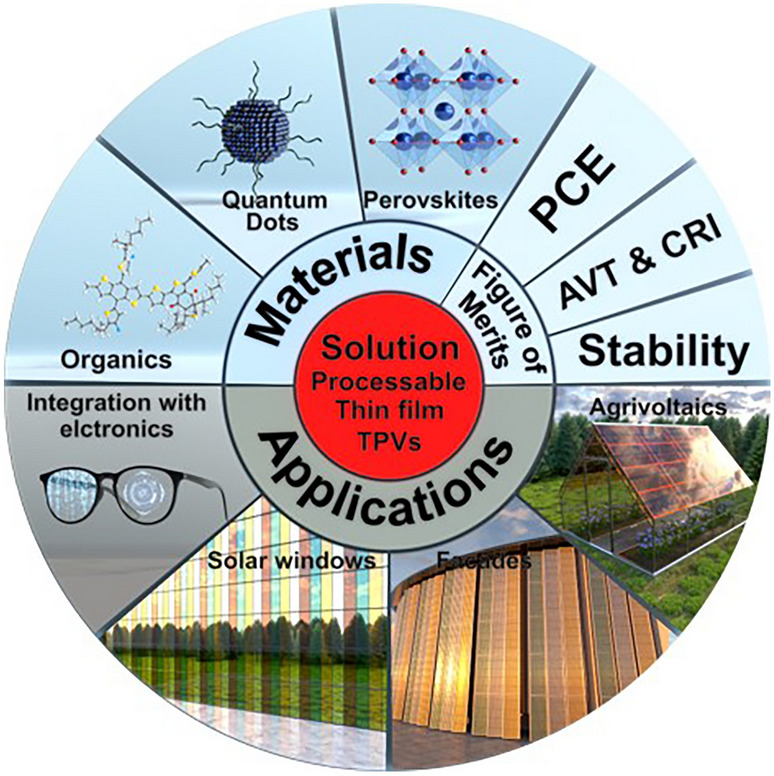

## Introduction

The ongoing economic expansion together with the growing awareness of how human activities are contributing to the climate change has triggered a surge of interest in renewable energy [1]. Among various renewable energy sources, solar energy is recognized as one of the most promising options for meeting future societal needs due to its ubiquity and abundance [2, 3]. Notably, the cost of conventional photovoltaic (PV) devices has markedly declined since the late-twentieth century, enabling both large solar farms and small house-scale power generation in remote areas. However, the space in dense urban environments is limited, which puts great difficulty in the solar panel installation. To overcome the spatial constraint, an idea of integrating PVs into building envelopes has been proposed [4–7]. However, this imposes extra requirements on aesthetics, leading to the concept of (semi-)transparent photovoltaics (TPVs).

The need for both renewable energy sources and buildings with aesthetic appearance in urban areas raises building integrated photovoltaics (BIPVs), which is one of the main applications and driving forces of TPV research. According to Salameh and coworkers’ simulation, a building with BIPV facades and solar windows in Sharjah in the United Arab Emirates had annually 27.69% less electricity consumption than that of the counterpart without BIPVs [8]. Anctil et al. applied a life cycle approach to evaluate the energy return on investment (EROI) of organic TPVs [9]. The organic TPVs used as skylight in Honolulu, Hawaii, showed an EROI of 208, indicating great energy and cost benefits. Indeed, some companies, such as Skanska and Saule, have already started field tests on emerging BIPV technologies [5].

While TPVs are promising energy harvesting devices in cities, the extra requirements on aesthetics make it more complex to evaluate their performance. As transparency and color put constraints on the amount of light absorbed, there is a trade-off between energy generation and aesthetics. Therefore, the figures of merit of conventional opaque PVs must be extended to include average visible transmission (AVT) and color rendering index (CRI) to correctly assess TPVs. However, it is still difficult to compare the efficiency of TPVs with different AVTs. In this context, Traverse et al. first proposed a compound figure of merit, light unitization efficiency (LUE), defined as the product of power conversion efficiency (PCE) and AVT [10]. LUE reflects not only device performance in terms of power generation and transparency, but also the trade-off relationship between them.

Currently, conventional silicon solar panels still dominate the global BIPV market, as they have already been well commercialized [5, 7]. Because crystalline silicon is opaque, the common way to enhance transparency is to increase the gap between micro-sized cells or create larger micro-holes within cells for light transmission, called spatial segmentation method. A typical silicon TPV can reach a PCE of 12.2% and AVT of 20% [11]. Meanwhile, since light is directly transmitted through gaps or micro-holes, spatial segmented silicon TPVs generally show a neutral color. However, there are several challenges in silicon-based BIPVs: (i) the fabrication processes become even more complex than the opaque silicon solar panels; (ii) transparency and aesthetics can be improved only at the great expense of device efficiency; (iii) it is hard to tune the color to meet the demand of the building facades. In addition to silicon TPVs, other conventional PV technologies, such as CdTe and Cu(In_1−*x*_,Ga_*x*_)Se_2_, can also be applied in transparent forms by reducing the thickness of light absorber [12, 13]. But they share similar drawbacks as silicon PVs, such as a low efficiency and charmless appearance. More importantly, the deposition of the conventional PV materials generally requires vacuum and high temperatures, which is much more expensive and complex than solution processing such as blade-coating and spin-coating.

These days, emerging PV materials such as perovskite [14–22], organic molecules [3, 23–27], and colloidal quantum dots (CQDs) [28–38] have attracted a great attention in both academic and industrial communities. These materials possess solution-processability and bandgap tunability, enabling a large-scale fabrication at a reduced cost and a feasible customization of both transparency and color. Furthermore, the last decade has witnessed a rapid development in perovskite solar cells (PSCs) that the PCE has boosted from 3.8% [39] to 26.7% [40] with the aid of solvent [41–43], additive [14, 44–47], composition [48–52], and interface [21, 53–59] engineering strategies. The performance of organic photovoltaics (OPVs) has also increased significantly in the last 10 years due to the intensive research in near infrared (NIR) absorbed polymer donors [3, 60, 61] and non-fullerene acceptors [27, 62–66]. In addition to molecular engineering of donors and acceptors, other strategies, including cathode interfacial materials [67], ternary blends [68, 69], layer-by-layer deposition [69, 70], and solid additives [71], were also developed to further push the efficiency of OPVs to over 20%.

These advancements in conventional opaque PVs shed light on how to further improve efficiency and transparency of the TPVs. For instance, the solvent and additive engineering developed in the opaque PSCs can be transferred to eliminate pinholes in the ultrathin perovskite films for the TPV application [72]. The methods to redshift an organic molecule absorption window in the OPVs can be used to reduce the visible light absorption in the organic TPVs to improve AVT and CRI [73]. As a result, the past few years have seen a rapid development in those solution-processed thin-film TPVs. In this context, Yu et al. and Kini et al. reviewed recent strategies used to improve the absorber layer in organic TPVs [74, 75]. Burgues-Ceballos et al. and Li et al. summarized the latest progress in organic TPVs in terms of BIPV applications [7, 76]. Bing and coworkers illustrated the potential of perovskite TPVs in glazing application [5]. Xiao and coworkers comprehensively reviewed the strategies for light management and transparent electrodes to enhance AVT and CRI [72]. Pulli et al. summarized the recent strategies of wavelength-selective absorption and analyzed a simulation case to demonstrate the economic and environmental benefits of TPVs [77]. However, most of the previous reviews focus on either one application or one type of TPVs. There are limited numbers of review papers summarizing the requirements of various applications and comparing the merits and drawbacks across different types of photoactive materials. Our review emphasizes the emerging TPVs with solution processability and provides a systematic comparison between perovskites, organics, and CQDs. In addition, we discuss the prospective applications of the emerging TPVs based on the materials characteristics and the application requirements on the aesthetics and energy generation.

In this regard, this review aims to update the rapid development in the emerging thin-film TPVs, demonstrate versatile TPV applications in daily life, and assess the pros and cons of the emerging materials according to the application requirements on the aesthetics and energy generation. First, we briefly introduce current strategies to achieve transparency in the TPVs and categorization. Second, we discuss the figures of merit of the TPVs and demonstrate the theoretical limits to provide comprehensive understanding of the current status of the technology. Third, recent progress in various TPVs is summarized with a particular focus on solution-processed thin-film TPVs, including perovskites, organics and CQDs. Fourth, we provide the prospective applications of TPVs with emphasis on agrivoltaics, smart windows and facades. Finally, current challenges and future opportunities in TPV research are highlighted.

## Transparent Photovoltaics

### Current TPV Technology

**Fig. 1:**
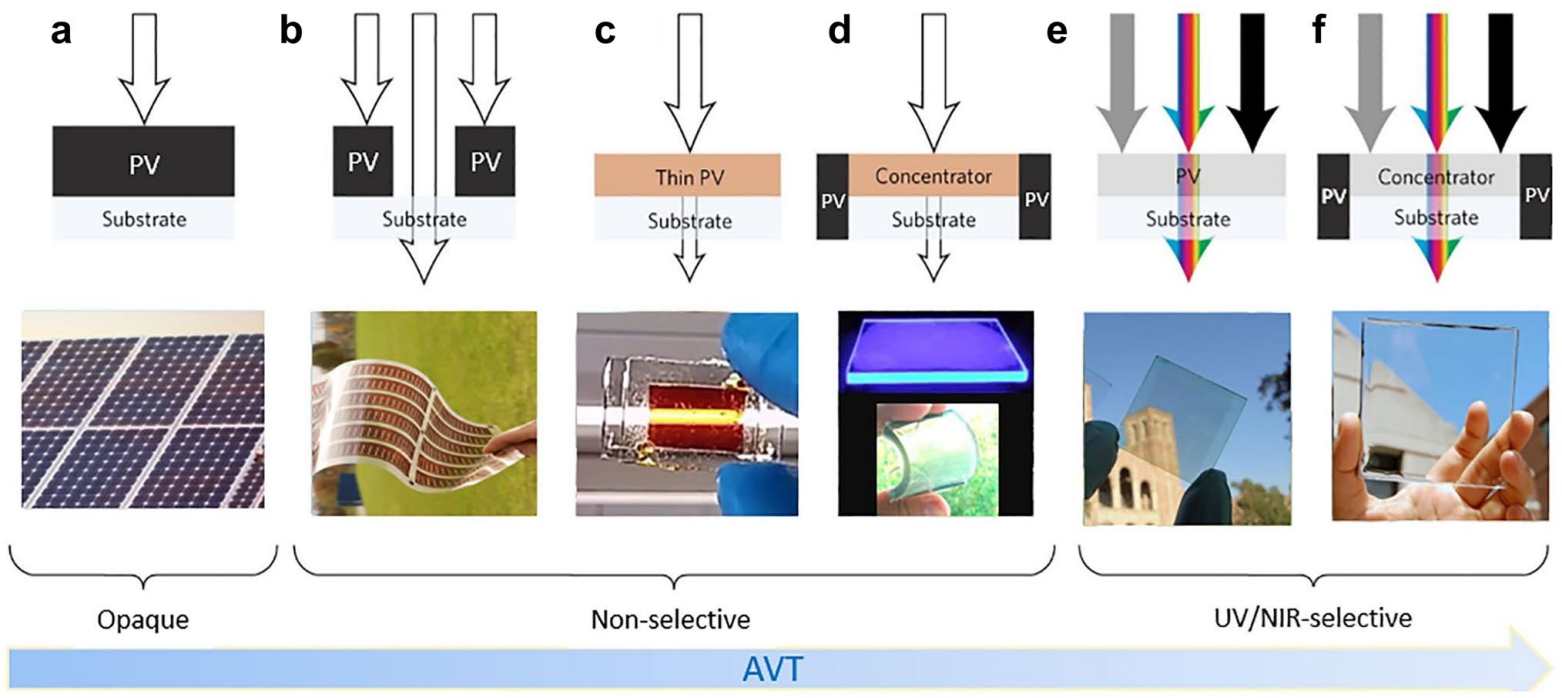
Schematics and real examples of **a** opaque and **b-f** transparent photovoltaics. Reproduced from Ref. [77]. Transparent photovoltaics may adopt **b-d** non-wavelength selective strategies or **e–f** wavelength-selective strategies

The fundamental idea of the TPV is to harvest light in only ultraviolet (UV) or NIR range or absorb light of all wavelengths but with a reduced amount. Largely there are two representative strategies as illustrated in Fig. 1a–f: (1) wavelength-selective and (2) non-wavelength-selective methods [77]. The non-wavelength-selective TPVs employ opaque photoactive materials in the form of spatial segmentations (Fig. 1b), thin films (Fig. 1c), or solar concentrators (Fig. 1d) to permit partial transmittance in the visible region. This method typically results in a lower AVT (0–50%) but a higher PCE (10%–15%) than those of the wavelength-selective TPVs [78, 79]. These features make the non-wavelength-selective technology promising in applications involving an outdoor decoration and colored glazing. In contrast, the wavelength-selective technology uses multi-junctions, organic molecules with discontinuous band structures **(**Fig. 1e), or solar concentrators (Fig. 1f) to selectively harvest UV and NIR light. As a result, the wavelength-selective technology allows a larger AVT (50%–90%) and a higher color rendering index (CRI), making it suitable for solar windows and agrivoltaics [80, 81].

As one of the most straight-forward non-wavelength-selective strategies, the spatial segmentation has been utilized in silicon PVs. The opaque cells are cut into small pieces with gaps between them to allow light pass through (Fig. 1b) [77]. Ideally, as the transparency increases with larger gaps, *V*_*oc*_ and FF remain constant while *J*_*sc*_ decreases due to the reduced effective area. However, either mechanical or laser cutting may cause damage on materials and thus lowering the PCE. In addition, the cutting technique is not economically favorable for silicon PVs.

Reducing the thickness of photoactive layer is another intuitive non-wavelength-selective strategy to achieve transparency (Fig. 1c). Thin films increase throughput and decrease the overall cost due to reduced material use and deposition time. However, careful optical design is generally required to boost device performance [82]. Optical designs applied in opaque thin-film PVs may inspire light management in thin-film TPVs. For instance, in opaque thin-film silicon PVs, an anti-reflection coating is typically combined with a front texture to induce Lambertian scattering and effectively trap light in the absorber. Judicious design of front texture can be applied in thin-film TPVs to enhance absorption in NIR region. Distributed Bragg reflector (DBR), composed of multiple layers of alternating materials with discrepant refractive indexes, is another common optical design in opaque thin-film PVs to enhance light absorption at certain wavelengths. Li et al. have adopted this design in organic TPVs to selectively recycle NIR photons by reflecting them back into the light absorber [83].

Another conventional TPV technology is luminescent solar concentrator (LSC) where light is first absorbed and re-emitted by fluorophores, and then harvested by the opaque PVs at the edges of the LSCs as shown in Fig. 1d, f. In spite of an additional energy conversion process in the LSCs, they have attracted intensive research interests attributed to their advantages such as structural simplicity, angle independence, and high defect tolerance [84–86].

In the past few years, many research groups have reported state-of-the-art thin film TPVs with advancements in various aspects, including bandgap optimization [87], film deposition [88], and light management [89, 90]. For example, Matteocci and coworkers adopted electro-optical simulations to determine the optimal bandgap and thickness of each layer in wavelength-selective TPVs [87]. As a result, the champion device achieved the PCE of 6.3% at the high AVT of 69.4%. As an example of progress in film deposition, Yue et al. demonstrated large-area uniform coating of FAPbBr_3_ perovskite through solvent engineering in wavelength-selective TPVs, resulting in a 10 × 10 cm^2^ module with the PCE of 5.55% and the AVT of 53% [88]. As for the progress in light management, Liu et al. proposed an aperiodic band-pass electrode to enhance transmittance in visible and reflection in the NIR range so that the AVT was improved from 29.7% to 46.8% with the similar PCE [89]. As a result, the LUE of the wavelength-selective TPV exceeded 5% for the first time in this report. A similar strategy was also applied in non-wavelength-selective CQD TPVs by Kim et al. The asymmetric MoO_x_/Au/MoO_x_ electrode showed a higher transparency than the symmetric structure, boosting the AVT to over 20% [90].

In spite of continuous progress in the thin film TPV technology, there are still various challenges to be solved. First, it is still difficult to optimize PCE under the constraints of both AVT and CRI. A more sophisticated simulation model is needed to facilitate a judicious material and device design according to a desired transmission spectrum. Secondly, there is inconsistency in extracting figures of merit from the TPVs. For instance, some AVT values were extracted in the range of 380–780 nm which is the visible region defined by ISO 9050:2003 [5], while other AVT values were extracted in the range of 400–800 nm [72] or by reading the maximum transmission in the spectrum [91, 92]. Thirdly, the current TPV technology is still faced with challenges in aesthetics. Although some have demonstrated the TPVs with high CRI indexes over 90 [93, 94], the high CRI indexes have been achieved at the expense of energy generation (PCE below 1%), which makes TPVs inapplicable in daily life. Last but not least, the emerging TPVs show poor long-term stability under light, moisture, and oxygen, which is a huge obstacle to commercialization. In addition, the long-term stability of other figures of merit such as AVT and CRI needs to be studied along with PCE to better evaluate the current TPV technologies.

### Factors Need to be Considered When Developing TPVs

Manufacturing of TPVs requires additional consideration on the PCE, AVT, aesthetics, and long-term stability, meaning that new figures of merit in TPVs need to be optimised. Here, we discuss these figures of merit and their relationships with each other to understand the current TPV technology and future development.

**Fig. 2:**
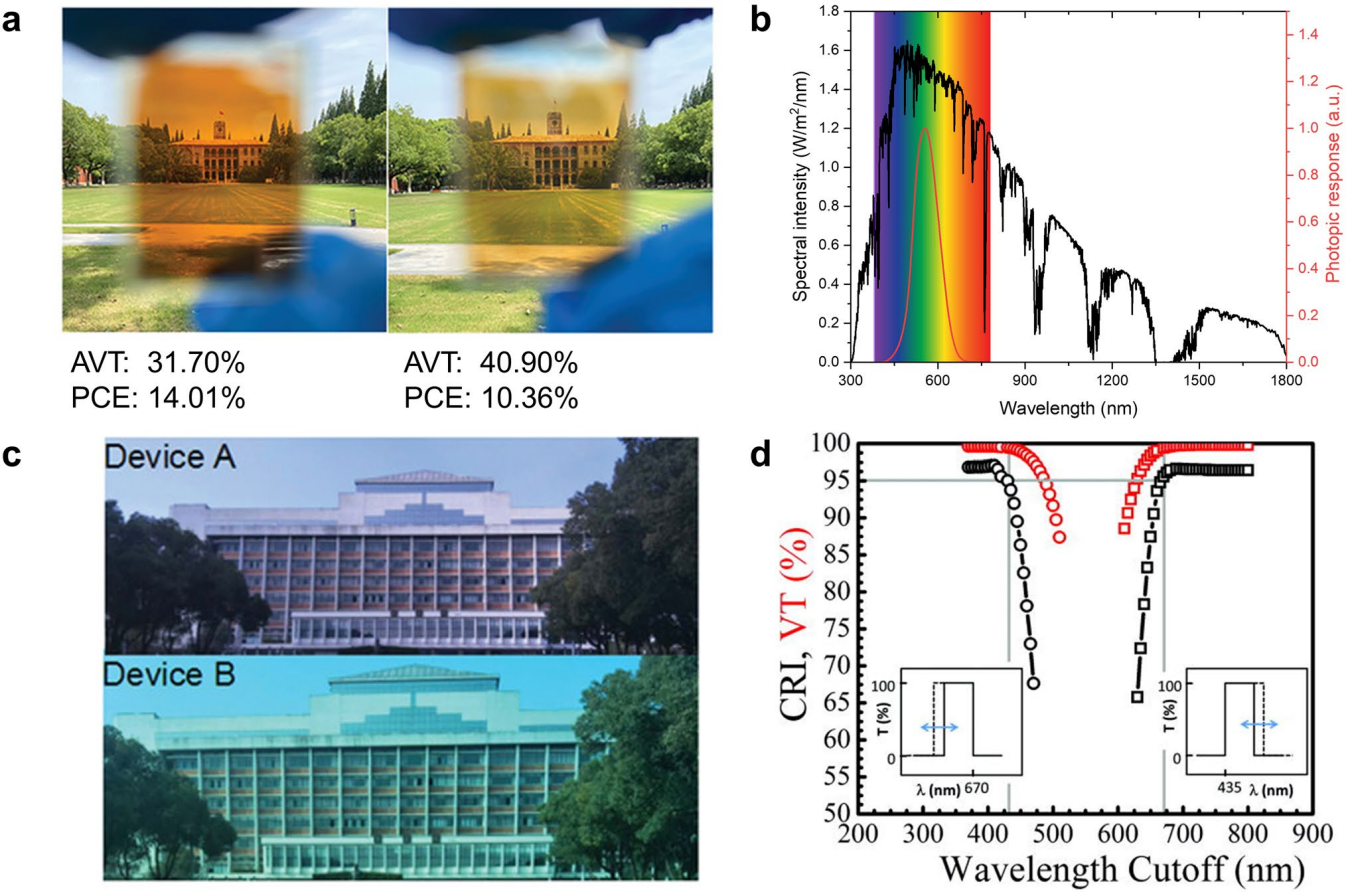
**a** An example intuitively showing the trade-off between PCE and AVT Reproduced from Ref. [95]. **b** AM 1.5G spectral intensity (black) and photopic response of human eyes (red). Visible region is highlighted in rainbow color. **c** Device A and B show similar transparency (an AVT of 22.35% and 23.45%) but distinct colors. Reproduced from Ref. [138]. **d** Theoretical CRI (black) and AVT (red) as a function of idealized transmission spectra shown in the insets. Reproduced from Ref. [100]

1. PCE and AVT: Except for the wavelength-selective TPVs, the higher transmittance in the visible region indicates less light absorption and a lower *J*_*sc*_. The decrease in *J*_*sc*_ usually results in a *V*_*oc*_ drop as well which further lowers the PCE. As a result, the non-wavelength-selective TPVs generally show a rapid drop in the PCE as the transmittance increases. Figure 2a intuitively demonstrates the trade-off between PCE and AVT, as the thickness of photoactive layers changes in non-wavelength selective TPVs [95]. Therefore, it is essential to evaluate and report the transmittance together with the PCE in the TPVs. The AVT is widely used to quantify transmittance and the definition is shown in Eq. (1) where the integral takes over the visible region, *λ* is the wavelength, *T* is the transmission, *P* is the photopic response, and *S* is the solar photon flux (AM1.5G) for window applications (Fig. 2b), or 1 for other applications.
  1 $$AVT = \frac{{\mathop \smallint \nolimits_{vis}^{{}} T\left( \lambda \right)P\left( \lambda \right)S\left( \lambda \right)d\lambda }}{{\mathop \smallint \nolimits_{vis}^{{}} P\left( \lambda \right)S\left( \lambda \right)d\lambda }}$$

In the case of the wavelength-selective TPVs, there is a less significant trade-off relationship between the PCE and AVT as the TPVs mainly harvest the UV and NIR light. The PCE of typical wavelength-selective TPVs could drop from 10.06% to 5.11%, while the PCE of non-wavelength-selective TPVs could decrease from 15.5% to 4.1% as the AVT increases from 20% to 50.8% [73, 96, 97].

To evaluate performance of the TPVs more comprehensively, LUE has been proposed, which is the product of the PCE and AVT [10]. In this way, the LUE can reflect the improvement in energy generation and transmittance as well as the trade-off between them, providing a method to compare the TPVs with various AVT values. To further improve the LUE, technical progress has to be made in photoactive materials, transparent electrodes, and optical coatings to reduce parasitic absorption while enhancing charge transport. In practice, the effect of environmental factors on the PCE and AVT may need to be considered as well. For instance, as the position of the sun changes on a daily and yearly basis, the PCE and AVT values of the TPVs are subject to change with time as well, which requires careful design and detailed analysis to balance efficiency and appearance [98, 99].

(2) Aesthetics: TPVs can be closely engaged in our daily life, leading to two important requirements on aesthetics: a proper AVT and CRI. In general, a sheet of glass with the AVT value above 60% looks clear while the AVT below 50% begins to look dark, colored, and reflective, which sets a boundary for the high and low AVT [10]. As TPVs with different transmittance spectra may share similar AVT values but in different colors as shown in Fig. 2c, a tint becomes another essential aesthetic requirement. Generally, the TPVs with a neutral color and high CRI are preferred as they do not distort the view behind them. This puts special requirements on the shape of the transmittance spectra. According to theoretical calculation performed by R. Lunt, complete transparency in the range 435–670 nm is enough to achieve an AVT over 99% and a CRI over 95 as shown in Fig. 2d [100]. This indicates the high absorption at the edge of the visible region has a small effect on the appearance of the TPVs, providing a guideline for the design of photoactive materials.
(3) Long-term stability: Since the merit of the TPVs lies in their integration with various real-life applications, the long-term stability becomes a critical factor for the evaluation of the TPVs. Ideally, the TPVs are required to operate for over the life expectancy of the product they are integrated with [5]. However, the current research on the TPVs has primarily focused on improving their PCE, while the long-term stability has received less attention. The thin film TPVs suffer from stability issues, such as moisture-induced degradation and photo-instability [6, 72, 74, 101, 102], which calls for further research. It is also worth noting that the stability challenge of the TPVs lies in not only PCE, but also AVT and CRI, which is different from the conventional opaque PVs. Although it is widely acknowledged that the transparency and tint are of the same importance as the PCE for the TPVs, the long-term stability in terms of the AVT and CRI has rarely been reported so far. Future efforts are needed to fill in this blank and establish a series of industrial standards for the commercialization.

### Theoretical Limits of TPVs

**Fig. 3:**
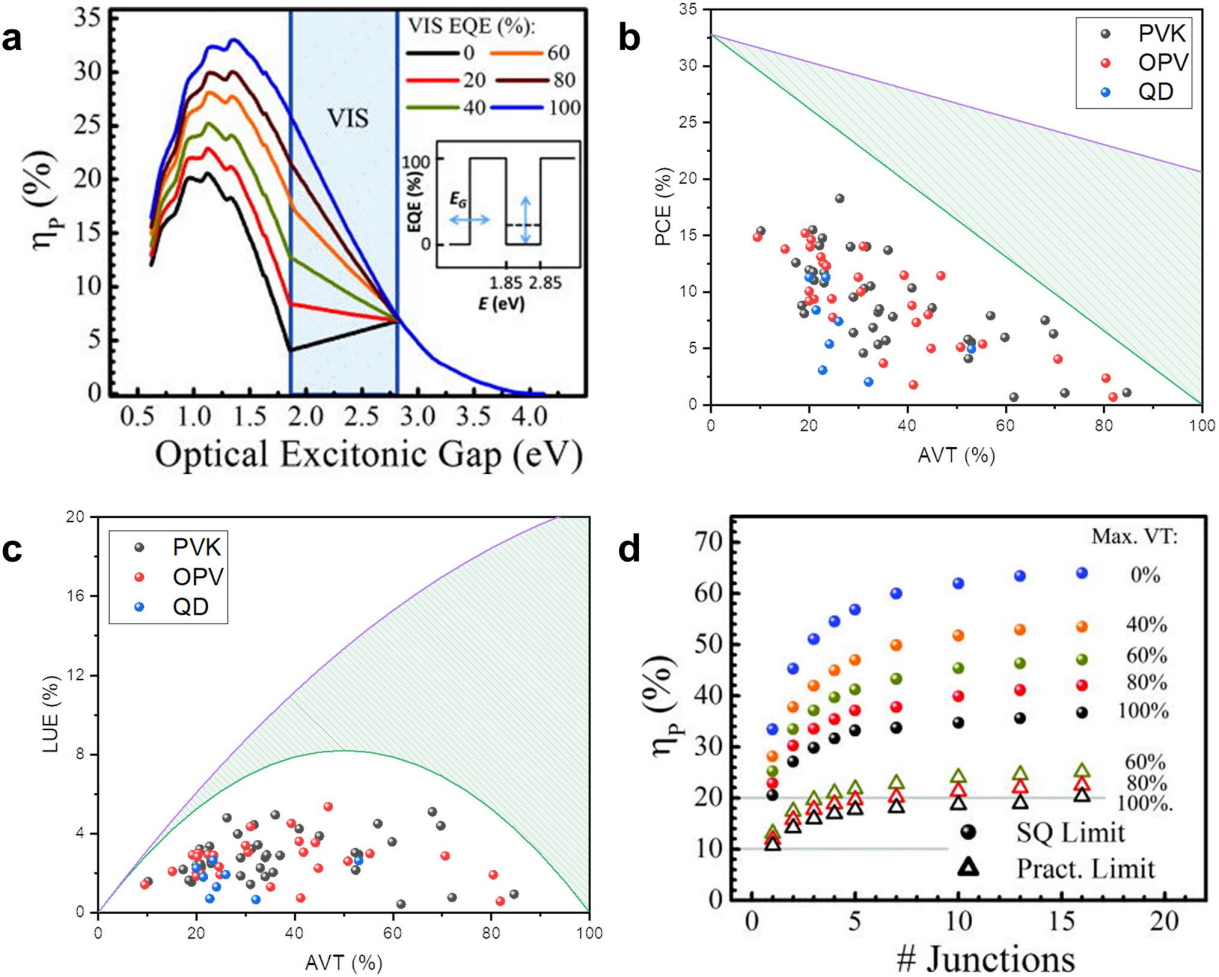
**a** Theoretical PCE limits as a function of optical excitonic gaps of single junction TPVs with different AVTs Reproduced from Ref. [100]. Scatter plots of recent advanced perovskite, organic, and CQD TPVs of **b** PCE vs AVT and **c** LUE vs AVT. Shockley Queisser limits of non-wavelength-selective and wavelength-selective strategies are shown in green and purple solid lines for reference. **d** Theoretical and practical PCE limits of series integrated multijunction TPVs with different AVTs. Reproduced from Ref. [100]

As 100% transmittance in the range of 435–670 nm is sufficient to achieve the AVT over 99% and the CRI over 95 [100], we narrow the visible region from 435 to 670 nm in the following discussion. It is worth noting that in AM 1.5 standard solar spectrum the UV (300–435 nm), visible (435–670 nm), and NIR (670–3000 nm) regions take up 8.6%, 35.0%, and 56.4% of energy flux, and 3.9%, 22.6%, and 73.5% of photon flux, respectively, indicating sufficient energy to harvest in UV and NIR regions. By assuming step-like EQE curves and applying the detailed balance model, known as Shockley–Queisser limit (S-Q limit), R. Lunt showed that the theoretical efficiency limit of a single junction solar cell dropped from 33.1% for the opaque PVs to 20.6% for the PVs with 100% AVT, and the optimal bandgap was redshifted from 1.36 to 1.12 eV as shown in Fig. 3a [100]. In contrast, in the ideal case for the non-wavelength-selective TPVs, the theoretical efficiency decreases to zero linearly with the AVT, showing the advantage of the wavelength-selective TPVs in applications requiring the high AVT, as shown in green shadowed areas in Fig. 3b, c.

A multi-junction structure is also valid for the TPVs to further enhance the PCE while maintaining the AVT. As the number of junctions reaches three, electron thermalization is considerably suppressed and the theoretical limit of the TPVs with an AVT of 100% increases to about 30%, as shown in Fig. 3d [100]. However, the multi-junction TPVs have a complex structure, and thus their price-to-performance ratio may not be as high as the single junction TPVs. In practice, when more limitations are considered, such as resistive loss, charge recombination, and parasitic absorption, we may assume 10% loss in *J*_*sc*_, 10% loss in FF, and 20% loss in *V*_*oc*_ compared to the S-Q limit, leading to 13% and 20% of PCE for the single-junction and the three-junction wavelength-selective TPVs, respectively.

**Fig. 4:**
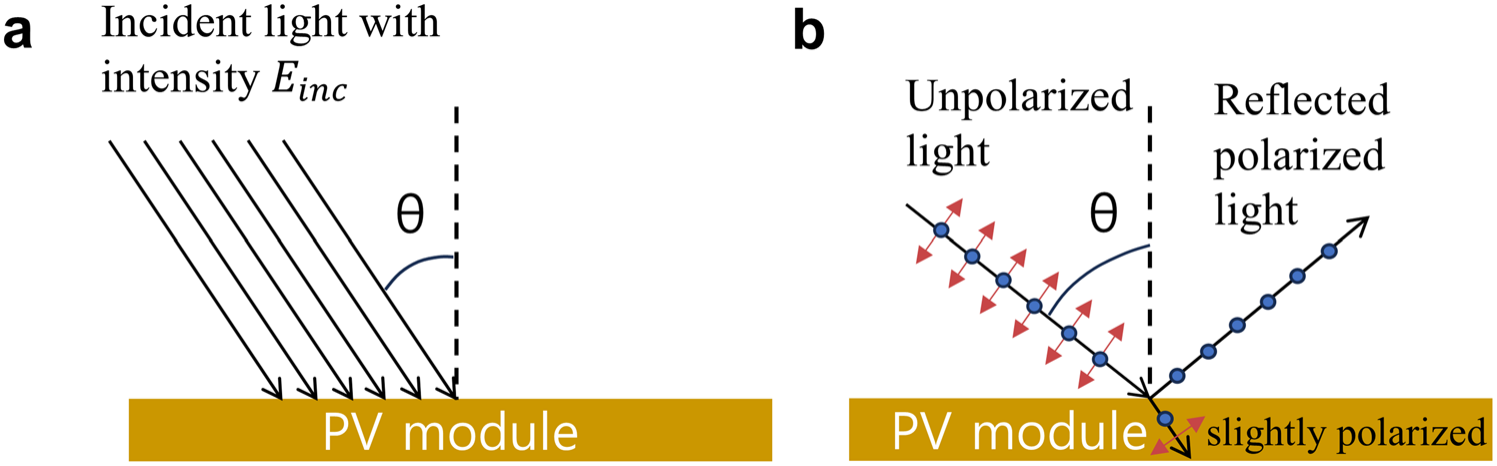
**a** Schematic of oblique incidence on a PV module with an angle θ. **b** Schematic of Brewster’s angle at which light with a particular polarization has no reflection. The red arrows indicate the polarization component within the paper plane while the blue dots represent the polarization component pointing out of the paper plane

However, the discussion above is based on normal incidence of AM1.5 sunlight. As the TPVs are integrated with other applications, it is difficult to always guarantee a normal light incident angle and no shade. Figure 4a illustrates oblique light incidence on a PV module with an angle *θ*. Consequently, the light intensity on the PV module is $E = E_{inc} \cos \theta$. The reduced light intensity generally causes a slight reduction in *V*_*oc*_ and FF and a main degradation in photocurrent such that $J_{sc} \left( \theta \right) < J_{sc} \left( {0^\circ } \right)\cos \theta$. Theoretical calculation via a transfer matrix method shows that the PCE degradation due to the angular response between 0 to 70° can be suppressed below 20% of the PCE under the normal incidence by optimizing the device configuration [103]. It is found that a high Brewster’s angle (Fig. 4b) can reduce the PCE degradation at large incident angles by allowing more transmittance of light with perpendicular polarization. According to Ding and coworkers’ simulation, with an improved angular response, the annual power generation of the BIPVs with the fixed angles could be enhanced over 30% [98]. The theoretical calculation indicates the need of designing TPVs with the limited PCE degradation up to 70° to maximize power output in a range of deployment conditions.

## Thin Film Transparent Photovoltaics

TPVs are one of the most promising technologies which meet the demand of energy and pleasing appearance in crowded urban areas. However, the TPV technology directly evolved from conventional PVs can not easily satisfy the requirements for customizing transparency and colors in real applications. In the past decade, researchers over the world showed great interest in the emerging thin film TPVs with tens- or hundreds-nanometer thick absorbers, including perovskite TPVs, organic TPVs, and CQD TPVs [7, 72, 104]. Attributed to the solution processibility and bandgap tunability of the light absorbers, these emerging thin film TPVs showed great advantages in the large scale manufacturing and color customization. However, each type of TPV has different challenges as the light absorbers get thinner, such as the non-uniform crystallization of perovskite [105], the low absorption of organic absorbers [106], and quantum dot degradation under ambient air [107]. The following sections will discuss these challenges and current strategies to overcome them in detail. The critical device performance of perovskite, organic, and CQD TPVs will be summarized in Tables 1, 2 and 3 respectively. Furthermore, the advantages and disadvantages of perovskite, organic, and CQD TPVs will be outlined in Table 4 for comparison.

### Perovskite TPVs

**Fig. 5:**
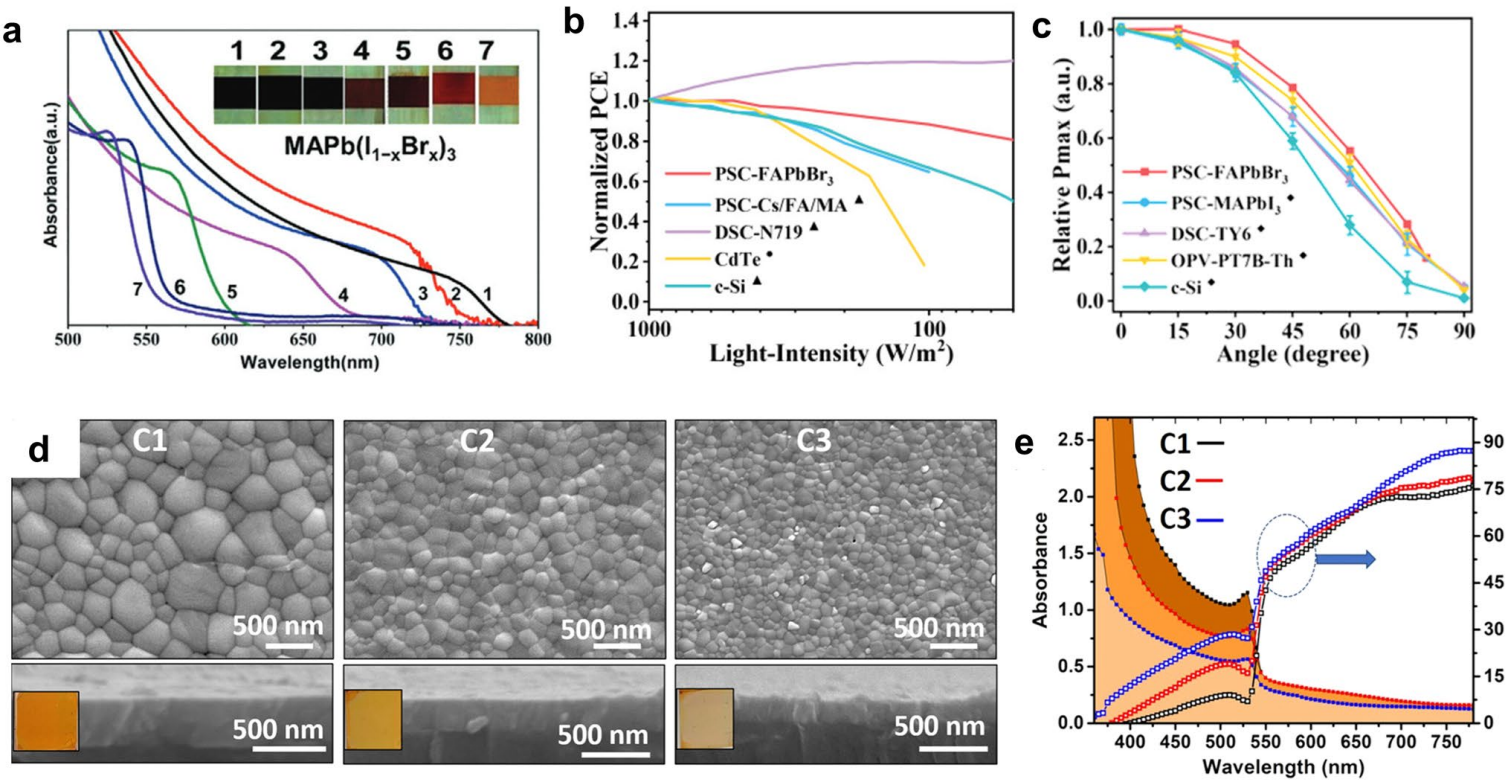
**a** Absorption spectra of PVK films with different compositions (x increases from 1 to 7). The inset figures show the color of the films. Reproduced from Ref. [108]. The dependence of PCE of different TPVs on **b** light intensity and **c** incident angle. Reproduced from Ref. [88]. **d** Morphology and film thickness of PVK films spin-coated with precursors at different concentrations (C1 > C2 > C3). Reproduced from Ref. [116]. **e** Absorption and transmittance spectra of the PVK films spin-coated with precursors at different concentrations. Reproduced from Ref. [116]

Lead halide perovskites, simply perovskites (PVKs) hereafter, are ionic materials with chemical formula APbX_3_ where A is a mono-covalent cation and X represents a halide anion. The bandgap of PVKs can be readily tuned by mixing halide anions or A-site cations, leading to different absorption spectra and colors as shown in Fig. 5a [108]. In addition, PVKs possess high charge mobility, large light absorption coefficient, and solution processibility, which are ideal for large scale PV applications [109, 110]. As a result, the last decade has already seen a significant breakthrough in the single junction opaque PSCs that the best PCE has exceeded 26% [111].

The rapidly increasing PCE of the opaque PSCs has also triggered research in PVK TPVs, especially for BIPV applications. Compared with other types of BIPVs, the PVK TPVs have the following advantages: (1) The large light absorption coefficient leads to high *J*_*sc*_ even in ultra-thin films [112–114]; (2) The bandgap tunability via composition engineering enables various AVT values and colors which are essential in applications with aesthetic requirements [6, 87]; (3) The solution processibility facilitates the large scale fabrication at a lower cost; (4) PSCs have a weak angle-dependence and intensity-dependence (Fig. 5b, c) [88], hence more suitable for the applications where solar angle and light intensity largely vary with time.

So far two main strategies have been developed to turn opaque PVK films into transparent ones. One strategy is to reduce the thickness of the PVK films [78, 93, 102, 112–114], while the other strategy is to enlarge the bandgap of the PVK films and selectively absorb the UV or blue light [87, 95, 115]. However, each strategy has its own challenges. For the thinner-film strategy, the main problem lies in rough morphology and low crystallinity of the films. While the thickness of the PVK film can be readily reduced by diluting the precursor, a low precursor concentration also leads to small grains and non-uniform crystallization (Fig. 5d, e) [116]. As a result, thinner films (< 200 nm) are prone to have more defects which act as non-radiative recombination centers and shunt paths. For the wider-bandgap strategy, one main challenge is the poor stability of PVK films due to phase separation. The challenge is rooted in the conflict between the need of a high Br/Cl doping ratio for the wide bandgap and the tendency of halide migration and segregation under light illumination due to the fragile ionic structure of PVKs. The other main challenge in the wider-bandgap strategy is the large *V*_*oc*_ loss in devices. Since most TPVs still use the charge transport layers designed for narrow-bandgap PVKs in opaque PSCs, there exists growing band mismatch as the bandgaps of PVKs increase, leading to a significant *V*_*oc*_ loss.

**Fig. 6:**
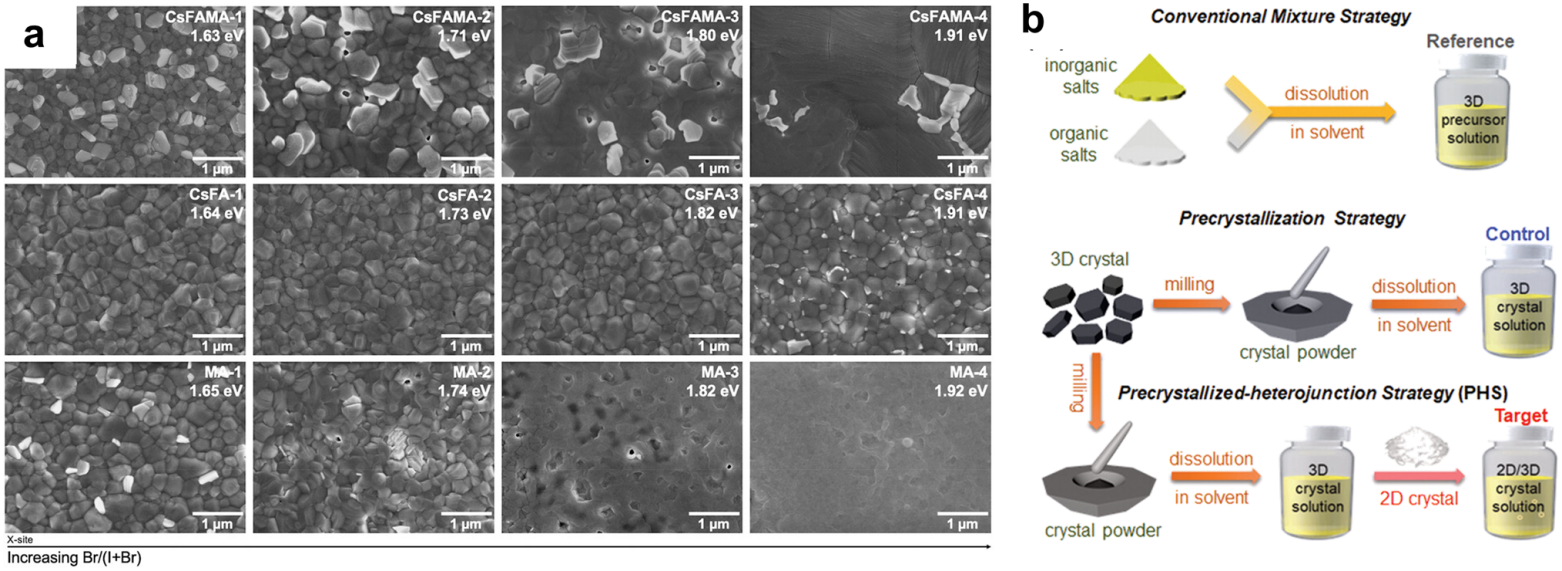
**a** SEM images of PVK films show phase segregation in MA-containing compositions rather than MA-free compositions. Reproduced from Ref. [97]. **b** Schematic diagrams show conventional strategy and pre-crystallization strategies to make precursor solution. Reproduced from Ref. [78]

To overcome the difficulties in the thinner-film strategy, researchers have developed composition engineering [79, 97, 117], additive engineering [95], and antisolvent method [116] to improve the morphology and crystallinity of PVK TPVs. For example, Yang et al. found that adding a small amount of MAPbBr_3_ (4 wt%) into MAPbI_3_ precursor could enlarge a size of colloids and reduce nucleation sites, leading to a uniform and larger grain size in ultra-thin films (Table 1) [79]. However, further increasing MAPbBr_3_ content resulted in lower device performance due to a phase segregation, requiring a precise control of the MAPbBr_3_ amount. Similarly, Yu et al. studied the effect of A-site and X-site composition on the PVK TPV performance and stability [97]. As shown in Fig. 6a, the authors reported that phase segregation was inclined to present in films with the MA-containing composition but not with the MA-free composition. In addition, aging of the MA-containing precursor solution resulted in a significant drop in the PCE due to the impurities formed during the MA degradation. Therefore, the authors concluded that a CsFA mixed cation composition is required for the high performance and stable PVK TPVs.

In addition to the composition engineering, incorporating additives is another common strategy used to improve the quality of PVK films. For example, Wang and coworkers introduced (chloromethylene)-dimethylammonium chloride (CDCl) into the diluted inorganic precursor solution to form intermediate adducts and assist crystallization [95]. As a result, they demonstrated all-inorganic CsPbI_2_Br TPV with the PCE of 14.01% and AVT of 31.7%, which outperformed PVK TPVs reported previously. Recently, Garai et al. applied tryptamine hydro bromide as the additive in Cs_0.1_FA_0.9_PbI_2_Br to enlarge grains and enhance crystallinity. With the aid of the additive, the TPV achieved a PCE of 14.21% at an AVT of 22.2% [118].

The employment of an antisolvent is another effective method to regulate the nucleation and crystallization. As the processes of the PVK nucleation and crystal growth are highly concentration-dependent, it is necessary to adjust timing for the antisolvent treatment when the precursor concentration is modified. In this context, Shivarudraiah et al. applied an in-situ photoluminescence (PL) measurement to determine the optimal timing for the antisolvent treatment for FAPbBr_3_ films [116]. It was found that the optimal antisolvent timing was at the beginning of the PVK nucleation while the antisolvent treatment earlier or later than the optimal timing could result in pinholes in the films.

Except for the three common methods discussed above, recently Zou et al. redissolved pre-crystallized 2D and 3D PVK crystals into precursors to improve the crystallinity and morphology of ultra-thin PVK films (Fig. 6b) [78]. The 2D PVK crystals not only acted as a nucleation center for a templated 3D PVK crystal growth, but also facilitated charge transport by forming an ideal energy landscape at grain boundaries. As a result, the PVK TPV achieved a PCE of 14.1% and an AVT of 22.1% with an active area of 1 cm^2^.

In an attempt to realize the large-scale fabrication of the PVK TPVs, Barichello and coworkers systematically examined the differences between a blade-coating and a spin-coating method in fabricating the structure of SnO_2_/FAPbBr_3_/Poly[bis(4-phenyl)(2,4,6-trimethylphenyl)amine (PTAA) [105]. While there was no noticeable difference in SnO_2_ and PTAA layers coated by the two different methods, a blade-coated PVK film exhibited thinner thickness, more pinholes, and larger surface roughness than those of a spin-coated PVK film. The poor quality of the blade-coated PVK film was due to the lack of control over the nucleation and crystal growth in the large area fabrication. As a result, PVK TPVs fabricated by the blade-coating method showed a lower PCE than that of the spin-coated ones.

**Fig. 7:**
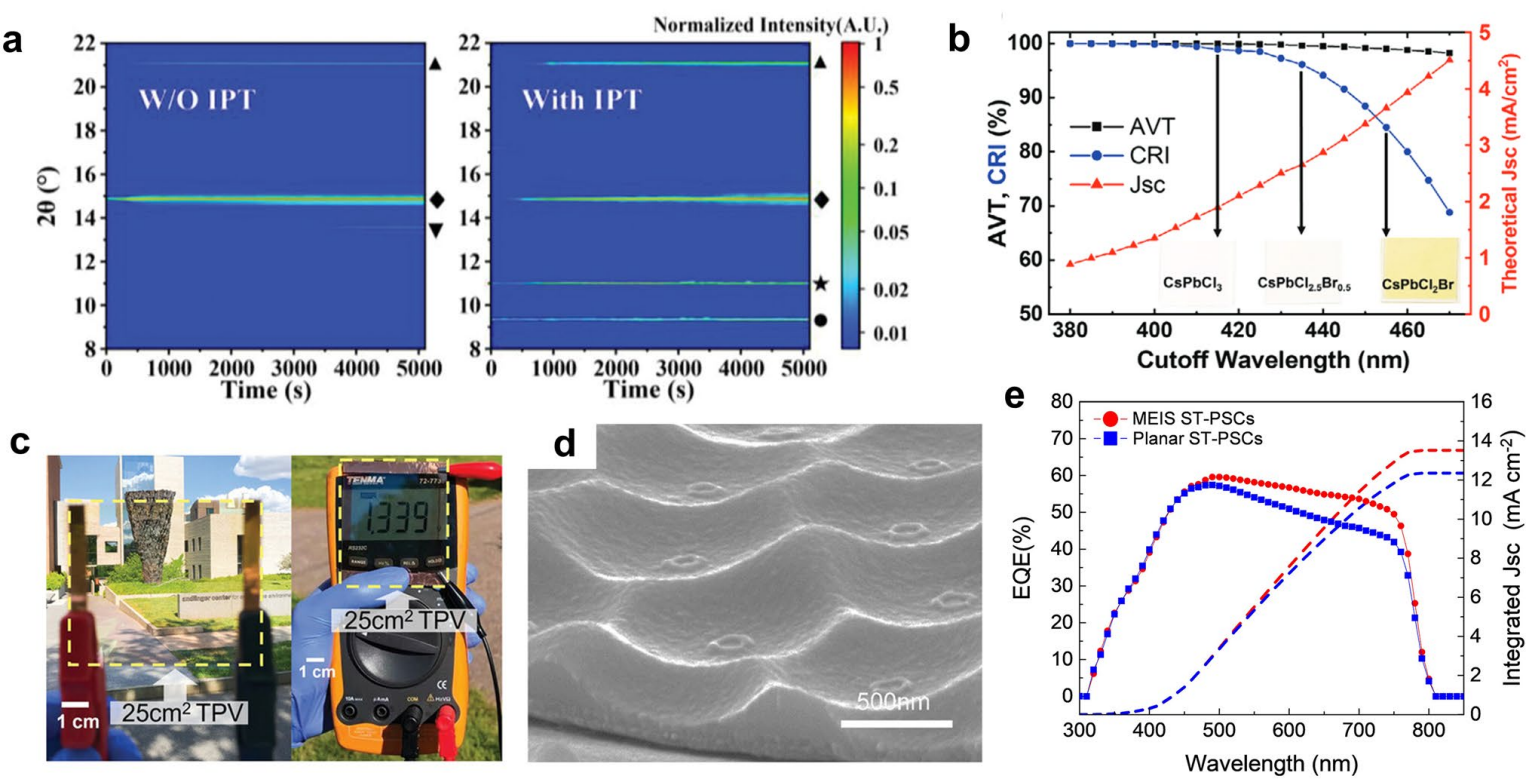
**a** In situ XRD false-color intensity maps of PVK films fabricated with and without intermediate phase transition (◆ and ▲ indicate FAPbBr_3_ PVK phase; ▼represents FAPbBr_3_-DMSO intermediates; ★ represents FAPbBr_3_-TMSO intermediates; ● represents PbBr_2_-TMSO intermediates). Reproduced from Ref. [88]. **b** Theoretical AVT, CRI, and Jsc limit calculated as a function of the absorption cutoff wavelength of CsPbCl_x_Br_3-x_ films (inset figures show the colors). Reproduced from Ref. [93]. **c** Photographs of a 25 cm^2^ TPV module with a high AVT of 84.6% and CRI of 96.5 without haze. The performance of the module was measured by a multimeter. Reproduced from Ref. [93]. **d** SEM images of SnO_2_/ITO with a moth-eye-inspired structure (MEIS) under a tilted-view. Reproduced from Ref. [120]. **e** External quantum efficiency curves of semi-transparent PSCs with and without MEIS. Reproduced from Ref. [120]

To gain a better control over the PVK crystallization in the large area by the blade-coating method, Wang et al. developed an intermediate-phase-transition (IPT) method by using a mixed solvent of DMF, DMSO, and tetramethylene sulfoxide (TMSO) [88]. As shown in Fig. 7a, an in-situ XRD measurement revealed that the addition of TMSO led to FAPbBr_3_-TMSO and PbBr_2_-TMSO intermediates, and retarded the nucleation by 3 s. This resulted in a 50% reduction in the crystal growth rate, and thus the formation of uniform and smooth films without pinholes. Using the IPT method, the authors demonstrated a PVK TPV module with a size of 10 × 10 cm^2^, and the module achieved a PCE of 5.55% and an AVT of 53%.

In addition, thermal evaporation is another facile method to deposit uniform ultra-thin films with arbitrary thicknesses [112], especially for wide bandgap PVKs in large scale fabrication [93, 102]. For instance, Roldán-Carmona and coworkers fabricated MAPbI_3_ films with thicknesses in the range of 40–280 nm by thermal evaporation [112]. The champion TPV with a PVK layer of 100 nm thickness showed the PCE of 6.41% with the AVT of 29%. Chen et al. introduced co-evaporation of CsBr and PbBr_2_ to fabricate an ultra-thin CsPbBr_3_ film as a wide-bandgap absorber for the tandem TPVs [102]. The authors found that keeping a stoichiometric ratio and a low evaporation rate (5 Å s^−1^) was critical to obtain films with high crystallinity and large grains. The stoichiometric ratio prevented the formation of defects and undesired phases, while the low evaporation rate facilitated adsorption of PbBr_2_:CsBr molecules on the substrate. As a result, the top TPV cell showed a PCE of 5.98% and an AVT of 59.8% with the high stability under the UV irradiation. However, the bandgap of CsPbBr_3_ (2.3 eV) was still not large enough to achieve a good color neutrality (Table 1).

The pursuit of higher CRI and transparency has spurred on research on the wide-bandgap PVK. For example, Liu et al. demonstrated a large-area (25 cm^2^) TPV module based on CsPbCl_2.5_Br_0.5_ which has the absorbance edge at 435 nm (Fig. 7b, c) [93]. Through the thermal evaporation of the PVK, the film showed a smooth and uniform morphology with the large area and achieved a high AVT of 84.6% and a CRI of 96.5 without haze. At the cost of aesthetics, however, the PCE reported was 1.1% due to the limited light absorption, indicating an important trade-off between the PCE and CRI in the ultra-wide-bandgap TPVs [117, 119]. To balance the PCE and CRI, researchers have utilized blue and green light partially by tuning PVK composition [87, 93]. Mateocci and coworkers theoretically calculated bandgap tuning with respect to composition tuning and designed the PVK TPV module based on MAPb(Br_0.87_Cl_0.13_)_3_. The module achieved a PCE of 6.3%, an AVT of 69.7%, and a CRI of 71.2 [87].

Except for tuning the bandgap of PVK, Zhu et al. proposed a light management strategy to enhance color neutrality [120]. Instead of reducing the absorption of green light, which human eyes are more sensitive to, the authors constructed a moth-eye structured TiO_2_ layer as shown in Fig. 7d. The structure could reflect more red light and thus the optical path of the red light was doubled. This strategy was able to enhance photocurrent and improve the CRI as the absorption over different wavelengths was balanced (Fig. 7e).

It is also worth noting that the *V*_*oc*_ loss tends to become larger in the TPVs adopting the wider-bandgap strategy (Table 1) [97, 102, 105]. While the PVK such as CsPbBr_3_ and FAPbBr_3_ possess bandgaps about 2.4 eV, their devices generally show a *V*_*oc*_ only about 1.4 V, indicating around 1 V of the *V*_*oc*_ loss, much larger than that in the opaque PSCs [102, 105]. One reason is that most wide bandgap PVK TPVs still use HTLs and ETLs that are designed for the opaque PSCs, leading to large misalignments in band structures. To be more specific, although the conduction band of PVK with a wider bandgap is higher in the band diagram, when electrons transport through ETLs, they lose more energy and drop to the energy level of the conduction band of ETLs [121]. It is the similar story for holes. Consequently, the *V*_*oc*_ of wide bandgap PVK TPVs is limited by the band positions of ETLs and HTLs. Another source of the *V*_*oc*_ loss lies in the quality of wide bandgap PVK films, as bromine- and chlorine-rich PVK are less frequently studied in the PSCs [122]. In addition, interfacial recombination plays an important role in the *V*_*oc*_ loss as well. Yuan and coworkers reported that the insertion of MXene (Ti_3_C_2_T_x_) between a SnO_2_ and PVK layer could passivate the interface and retard the crystallization process to form larger grains [123]. Consequently, the PCE was boosted from 12.37% to 14.78% while the AVT was maintained at 22.68%. Sharma et al. passivated the Cs_0.1_FA_0.9_PbI_2_Br /PCBM interface with alkylamine hydrochlorides to suppress the *V*_*oc*_ loss below 0.5 V [124]. The authors investigated the effect of carbon chain length on interface passivation and discovered that 4-chloropropylamine hydrochloride delivered the best performance. Recently, Girolamo and coworkers also applied alkylamine hydrochlorides to passivate the top interface in FAPbBr_3_ TPVs [125]. The device showed an ultrahigh *V*_*oc*_ of 1.73 V, indicating great importance of interface passivation for wide bandgap PVK TPVs. With further aid of anti-reflecting coatings, the TPV reached a record LUE of 5.72%.

Another challenge in the wide bandgap PVK TPVs is poor stability due to a photo-induced phase segregation, as the wide bandgap is usually achieved by incorporating Br^−^ or Cl^−^. To avoid the phase segregation, the doping ratio is generally limited [79]. Quasi-2D perovskites, Q2D PVKs hereafter, may provide another technical route to achieve a wide bandgap without the phase separation because the bandgap is tuned through quantum confinement [126]. In addition, the Q2D PVKs are more water-resistant and stable due to their hydrophobic organic spacers. However, most of the Q2D PVK films reported in literature have mixed phases with a large amount of a 3D-like phase, leading to a decrease in the bandgap and undesired absorption in the visible region. To date, there have been only a few reports on phase pure Q2D PVK films [127, 128] and no reports on Q2D PVK TPVs. Table 1 provides a summary of the literatures mentioned above.

The discussion above is focused on the PVK layer only. However, different from the conventional opaque PSCs, a transparent top electrode also plays an important role in TPVs [72, 129]. An ideal transparent top electrode has three requirements: (1) high transmittance; (2) low sheet resistance; and (3) mild deposition process which does not damage the underlying PVK and charge transport layer. Here we give a brief discussion about recent studies and more details can be found in a recent review on the transparent electrode [72]. Currently, there are four main categories of the transparent top electrode—transparent conductive oxides (TCOs), ultra-thin metal electrodes (UTMs), dielectric/metal/dielectric (DMD) electrodes, and nanomaterials-based electrodes such as Ag nanowires (AgNW). TCOs are known for their high conductivity and transmittance. However, they are usually deposited via magneton sputtering, which could damage the fragile PVK underlayer. One solution is to lower the sputtering power and deposit a buffer layer such as ZnO [130] and MoO_x_ [120] before sputtering to protect the layer beneath and reduce interfacial defects. UTMs are usually deposited via a thermal evaporation method, which has a negligible effect on the underlayer. However, the thickness should be below 20 nm to guarantee transparency, leading to the poor conductivity and discontinuous morphology [72]. A similar trade-off also presents in the nanomaterial-based transparent electrodes. DMD electrodes are very similar to the UTMs in terms of conductivity and mechanical flexibility. However, the DMDs offer precise light management via tuning the thickness of dielectric layers, which is an advantage over the UTMs [131]. In addition, the bottom dielectric layer may also enable continuous growth of the ultrathin metal layer and prevent metal diffusion and reaction with the PVK layer beneath.

### Organic TPVs

Organic TPVs have a number of advantages, such as flexibility, light weight, solution processability, and tunable absorption spectra, which provides great potential in BIPV, solar windows, and agrivoltaics applications [76]. Due to difficulties in tuning absorption spectra of fullerene acceptors (FAs) and the wide bandgap nature of donors, however, the organic TPVs could utilize only UV–vis light in their early development stage, resulting in challenges for balancing the PCE and AVT [75]. As discussed in Sect. 2.3. Theoretical limits of TPVs, the optimal bandgap redshifts in the case of the TPVs so that more photons in the NIR region can be harvested while maintaining the transmittance in the visible region. To this end, researchers developed low bandgap (LBG) donors and non-fullerene acceptors (NFAs) whose absorption window can be shifted into the NIR region [3]. As a result, the performance of the organic TPVs has been improved from a PCE less than 1% with an AVT of 26% to the PCE over 11% with the AVT of 46.8% (Table 2) [89, 132]. Meanwhile, discontinuous band structures of the organic molecules provide a unique opportunity to flatten absorption spectra in visible region while selectively harvesting UV and NIR photons. In contrast, semiconductors with continuous direct band structures, for example, PVKs, generally show rapidly increasing absorption spectra above the bandgaps. As more light is absorbed at shorter wavelengths in PVK TPVs, the only way to achieve high CRI is to give up most of the visible light, leading to a low device efficiency. However, the selective absorption offers organics a great advantage over PVKs in TPVs, as it provides a method to simultaneously obtain a high PCE over 8% and an excellent CRI near 100 [94]. Application-wise, organic TPVs with high CRIs can be directly attached to building envelopes as BIPVs without worrying about the appearance. Also, thanks to the flexibility of organics, organic TPVs can be readily installed on complex curved surface of modern architecture and exhibit a smaller degradation in PCE than conventional silicon PVs when subjected to the same mechanical strain [133]. In addition, unlike PVK TPVs, the thickness of the organic TPVs can be easily controlled by changing precursor concentrations and speeds of spin-coating without affecting the quality of the films.

However, organic TPVs are faced with other challenges. First, the PCE of the organic TPVs is still lower than PVK TPVs at the same AVT in many cases [78, 116, 120, 134, 135]. Although theoretically the organic TPVs are able to achieve both high AVT and PCE by selectively harvesting UV and NIR light, the current PCE of the organic TPVs still lags behind the non-wavelength-selective PVK TPVs even in the case of high AVT > 50% (Fig. 3b, c) [80, 87, 93, 96]. Second, it is still difficult to achieve high AVT and CRI values in organic TPVs. Although various strategies have been developed to red-shift absorption peaks into the NIR region, the photoactive layers inevitably possess non-negligible uneven absorption in the visible region, which limits both transparency and color neutrality. In addition, the conventional bulk heterojunction device structure has a complex interface which significantly scatters light and further reduces AVT.

**Fig. 8:**
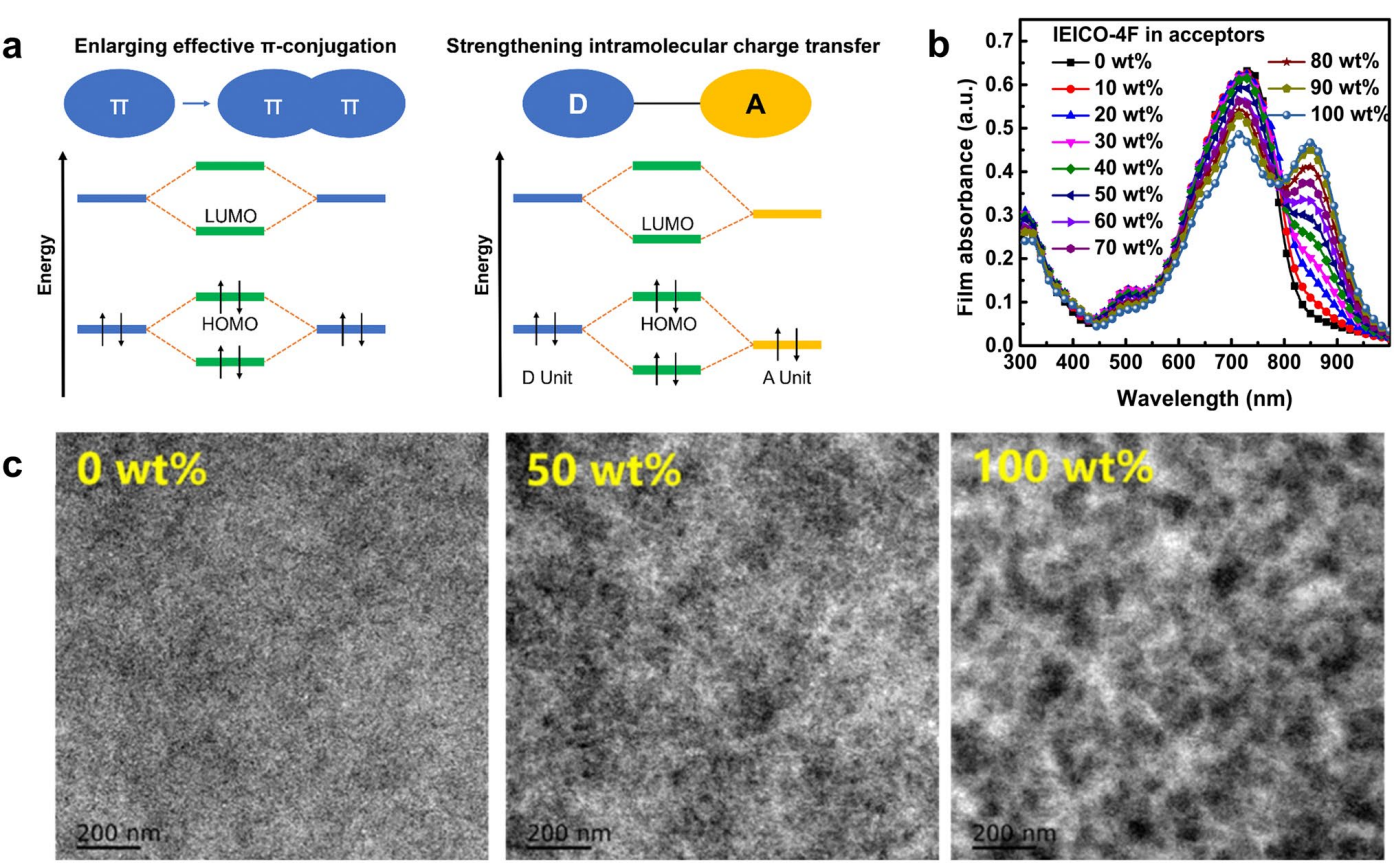
**a** Schematics showing two common design strategies, inducing intramolecular charge transfer and extending conjugation length, to lower bandgaps of organic molecules. Reproduced from Ref. [74]. **b** Absorption spectra of ternary blend films with different IEICO-4F content in acceptors. Reproduced from Ref. [139]. **c** TEM images of IEICO-4F:BDTThIT-4F:PTB7-Th ternary blend (0, 50, and 100 wt% of IEICO-4F in acceptors). Reproduced from Ref. [139]

Regarding the challenge of low PCE, one possible solution is to further redshift the absorption window of the acceptors and donors into the NIR region. This can be achieved via various molecular engineering strategies [3, 75, 96, 136], such as inducing stronger intramolecular charge transfer and extending conjugation length as illustrated in Fig. 8a [74]. Recently, Huang and coworkers introduced a new strategy by replacing S with Se in heterocycles, and the absorption peak of the new acceptor redshifted by 54 nm [73]. As a result, the PCE of the opaque device increased from 12.4% to 13.2% while the transparent device reached the PCE of 10.1% with the AVT over 20%. In another work, Wei et al. tuned the regioregularity of polymer donors and found that the polymer (66-PTB) with a more planar backbone showed extended conjugation, enhanced internal charge transfer (ICT), and therefore a narrower bandgap [96]. Consequently, the 66-PTB-based TPV reached the PCE of 5.11% with the AVT over 50%. Duan and coworkers recently demonstrated that solid additives could also regulate the absorption spectra [137]. The authors employed 1,3-diphenoxybenzene as the additive to broaden the absorption of PP2:BTP-eC9 blend and achieved a PCE of 11.1% at an AVT of 43.0%.

Another popular method for enhancing the PCE is to apply a ternary photoactive layer. The third component is added to tune absorption spectra [94, 138–140], increase phase purity [141], and manipulate morphology [139]. For instance, Hu et al. reported that the addition of IEICO-4F into PTB7‐Th: BDTThIT‐4F could enhance light absorption in the NIR region (Fig. 8b) while the addition of BDTThIT‐4F into a PTB7‐Th:IEICO-4F blend could form nano-fibril phases with high purity rather than large aggregated phases (Fig. 8c) [139]. Therefore, the ternary organic TPV showed both high FF and *J*_*sc*_ compared with the two binary cases. Similarly, Wang and coworkers added BTTPC into a PBDB-TF:Y6 blend and found that a hole mobility and exciton dissociation were improved due to the fibrous phase aggregation [138]. As the three components all showed absorption peaks in the NIR region, the PCE was improved while the transmittance peak was tuned to fit with the photopic response of human eyes. In another work, Albab combined a PM6:Y6:PCBM ternary blend with self-assembled monolayers at both ITO/ZnO and ZnO/ternary blend interface to facilitate charge transport [142]. The resulting organic TPV showed a PCE of 10.37% and an AVT of 36.3% with the help of a MoO_3_/Ag/MoO_3_ electrode.

The organic TPVs are also faced with challenges in optical properties which are rooted in both materials and device structure. On one hand, although that various organic materials with absorption peaks in the NIR region have been developed, uneven absorption in the visible region is still inevitable, lowering the CRI of the organic TPVs. On the other hand, the conventional bulk heterojunction (BHJ) structure used in the organic TPVs causes significant light scattering at the interface and puts a constraint on the donor–acceptor (D–A) ratio, resulting in the limited AVT.

**Fig. 9:**
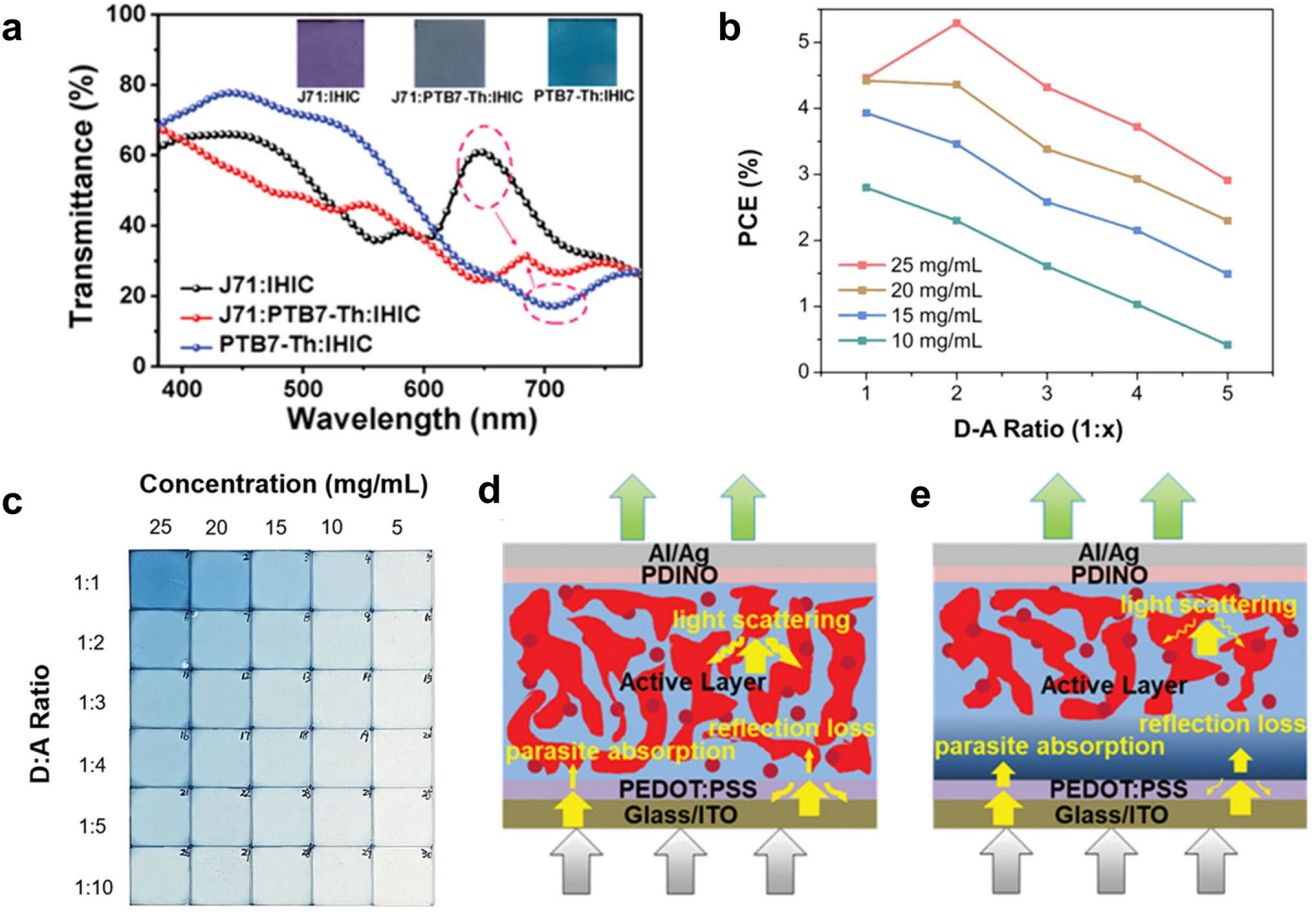
**a** Transmittance spectra of binary and ternary blend films with images as insets. Reproduced from Ref. [94]. **b** Images of BHJ films with different thicknesses (i.e. precursor concentrations) and D-A ratios. Reproduced from Ref. [80]. **c** PCE of TOPVs fabricated with different D-A ratios and precursor concentrations. Reproduced from Ref. [80]. Schematics of light interaction with **d** conventional BHJ device structure and **e** recently proposed PPHJ structure. Reproduced from Ref. [106]

In order to improve the CRI in the organic TPVs, one of the conventional strategies is to widen the bandgap of the organic materials. However, the high AVT and CRI were achieved at the cost of the low PCE [81, 143]. Recently, Zhang et al. combined the broad absorption spectra of the ternary strategy and a dielectric mirror to flatten the transmittance spectra in the visible region as shown in Fig. 9a [94]. As a result, a CRI near 100 was achieved in the organic TPV with the PCE of 9.37%. As more visible light was absorbed to flatten the transmittance spectra, however, the AVT was limited to 21%. In another work, Xu and coworkers adopted a different approach where the absorption windows of photoactive materials were further redshifted in the NIR region rather than designed to compensate with each other in the visible region [144]. In this work, a new small molecule acceptor, BZO-4Cl, was synthesized based on Y11, and the device exhibited the high CRI of 90.67 and the PCE of 9.33% at the higher AVT of 43.08%.

In addition to materials engineering, light management is another strategy to achieve high color neutrality in organic TPVs [145]. As organics typically show relatively weak light absorption, distinct methods to modulate light such as an optical coupling layer [146], micro-cavity [91], Fabry–Perot electrode [92], and outcoupling architecture [83] have been developed to enhance the visible transmission and reflect the NIR light back into the cell [145].

To further improve the AVT, one of the strategies is to reduce the amount of donors which typically possess an absorption window partially within the visible region. Li et al. diluted a donor with visible absorption and applied alloy-like NIR acceptors to further push the AVT from 32.3% to 39.3% [147]. Meng and coworkers investigated the influence of a donor–acceptor (D–A) ratio on the organic TPV performance. They found that though a low donor content could improve the AVT, the device efficiency dropped significantly due to poor charge separation and transport as illustrated in Fig. 9b, c [80]. To overcome the challenges resulting from the low donor content, Jing and coworkers introduced [2-(9-H-Carbazol-9-yl) ethyl] phosphonic acid (2PACz) as an additive into the photoactive layer [148]. 2PACz formed a self-organized layer and acted as a hole selective layer which effectively enhanced exciton dissociation and charge transport. A new device structure, called pseudo planar heterojunction (PPHJ), has also been proposed to solve the issue from the D–A ratio in the BHJ [24, 106, 134, 149]. The new structure could reduce the D–A interface so that the light scattering and parasitic absorption were reduced as shown in Fig. 9d, e. It was found that the strategy was effective to a number of binary systems and even ternary systems [106]. In addition, the thickness of the donor and acceptor could be tuned independently with only a small impact on the exciton dissociation at the D–A interface [134, 149]. Consequently, the transmittance spectra can be finely tuned without the limitation set by the D–A ratio. The performance of transparent OPVs mentioned above is summarized in Table 2.

### CQD TPVs

CQDs are semiconducting nanocrystals with diameters typically under 20 nm. Their bandgap increases as their size decreases, allowing size-tunable optoelectronic properties. CQDs are particularly advantageous for the PVs due to their ability to harvest a broader range of the solar spectrum compared to PVK and organic materials [150–153]. The high spectral tunability of CQDs is crucial for the fabrication of the semi-transparent CQD PVs, providing essential transparency in the visible range along with the high absorption in the NIR spectrum [154]. In fact, many CQDs, such as PbS, PbSe, and CdTe show strong absorption in NIR region [33, 155, 156]. For instance, the bandgap of PbS CQDs can be tuned from 0.4 to 1.5 eV so that light absorption can go beyond 2000 nm. As the optimal bandgap redshifted to 1.12 eV for TPVs, the ability to selectively absorb infrared light is essential to achieve both high efficiency and transparency. Through compositional engineering, CQDs can also be tailored to offer versatile color customization, merging an aesthetic appeal with a functional energy generation in applications such as BIPVs. Moreover, the inherent compatibility of the CQDs simplifies their incorporation into diverse systems, offering significant potential for innovations in the solar window technology [157].

**Fig. 10:**
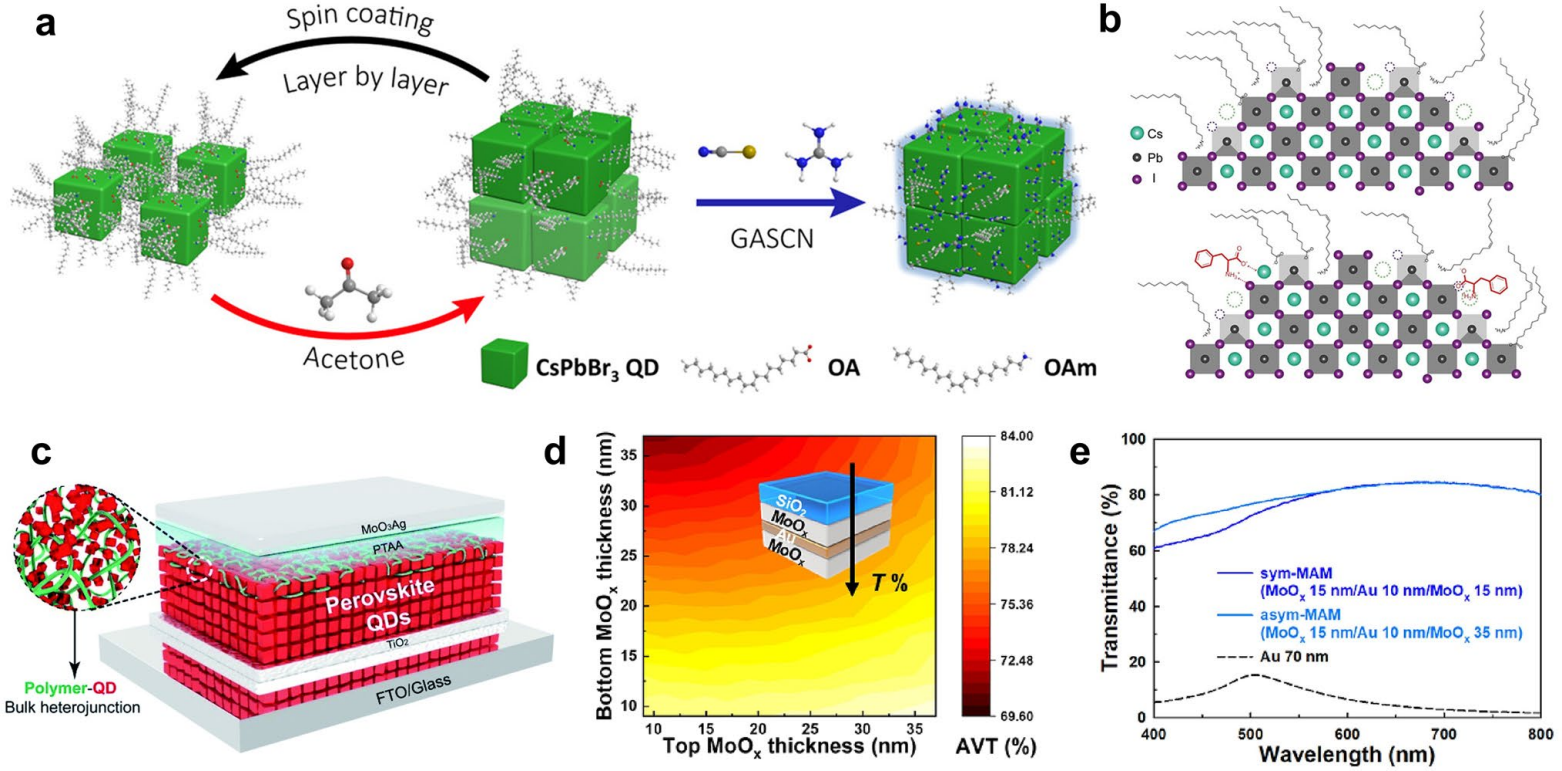
**a** Schematic illustration of CsPbBr_3_ QD layer deposition and ligand exchange Reproduced from Ref. [104]. **b** CsPbI_3_ QDs with and without phenylalanine (L-PHE) surface modification. Reproduced from Ref. [159]. **c** An example of QDs embedded in a polymer matrix to form a BHJ. Reproduced from Ref. [163]. **d** AVT of an asymmetric MAM structure as a function of the thicknesses of bottom and top MoO_x_ layers and **e** transmittance spectra of symmetric and asymmetric MAM structures. Reproduced from Ref. [90]

Efforts have been directed at optimizing the design of CQD layers to enhance the PCE under the low-light conditions. A solution-phase ligand exchange has proved to be an effective method to increase a CQD packing density and minimize energetic heterogeneity, thereby increasing photocurrent without compromising a *V*_*oc*_ [158]. Leveraging this method, Zhang et al. achieved the PCE of 8.4% in the CQD TPVs, along with the AVT of 21.4% (Table 3) [107]. This device also demonstrated improved photostability, retaining 85% of the initial PCE after 540 h of continuous illumination at standard 1 Sun condition. CsPbBr_3_ CQDs, which have larger bandgap compared to PbS QDs, have also been exploited as a light absorbing layer for the CQD TPV and demonstrated an excellent transmittance beyond the wavelength of 500 nm. The replacement of long-chain insulating ligands with guanidinium thiocyanate salt has improved the PCE, resulting from the enhanced surface passivation and charge transport (Fig. 10a) [104].

Enhancing the stability of CQDs is another crucial challenge for BIPV applications since exposure to ambient air can lead to the degradation of the CQDs. For instance, CsPbI_3_ CQDs can undergo an undesired transformation into the δ-phase in humid conditions, while exposure to oxygen can increase defect density in PbS CQDs. Recent studies have addressed this challenge through refinements in material compositions, modifications of the surface ligands (Fig. 10b) [159, 160], and the incorporation of CQDs into a host medium (Fig. 10c) [161–163].

Efforts to transition the traditional architecture of the CQD devices to the TPVs have also included the development of semi-transparent electrodes. For example, an Au electrode, one of the commonly used electrodes in CQD PVs, has been engineered for high transparency and conductivity. In 2016, Zhang et al. developed the first semi-transparent CQD PV with a thin PbS CQD layer and a 10 nm transparent Au back contact, achieving a PCE between 2.04% and 3.08% and an AVT from 32.11% to 22.74% [164]. However, the ultrathin Au electrode, despite its transparency and ease of the deposition, caused a substantial photocurrent loss due to a poor light reflection at the metal-CQD boundary and inadequate CQD absorption. Attempts to decrease the thickness of the Au electrode further led to the increased surface defects and electron scattering, which hampered the device performance without significantly improving the AVT [165]. To overcome these issues, a nanolayered MoO_3_/Au/MoO_3_ (MAM) electrode was introduced [166]. This sandwich structure enhanced the transparency of the electrode and minimized an internal optical loss, resulting in a PCE of 5.4% and an AVT of 24.1%. Further innovations led to the development of an asymmetric MAM structure with a thicker top and thinner bottom MoO_3_ layer (Fig. 10d), which effectively reduced the parasitic absorption within the MAM stack, thereby boosting the transmittance of the electrode (Fig. 10e) [90]. Applying this strategy to CsPbI_3_ CQDs yielded a PCE of 11.3% with an AVT of 23.4%.

Beyond the Au electrode, exploration into cost-effective alternatives for the TPVs is underway. Dastjerdi et al. utilized a cost-effective Cu electrode coupled with poly(3-hexylthiophene-2,5-diyl) as a hole transport layer to precisely adjust the valence band energy, achieving a PCE of 7.4% with AVT of 26% [167]. Moreover, Tavakoli et al. fabricated semi-transparent CsPbI_3_ CQD TPVs using a graphene electrode, which is chosen for its exceptional conductivity and superior transmittance [168, 169]. This strategy elevated the AVT to 53%, although the PCE was compromised, representing a notable advancement towards enhancing the transparency of the devices for various practical applications. The device performance and strategies applied in the CQD TPVs mentioned above are summarized in Table 3.

## TPVs Applications

**Fig. 11:**
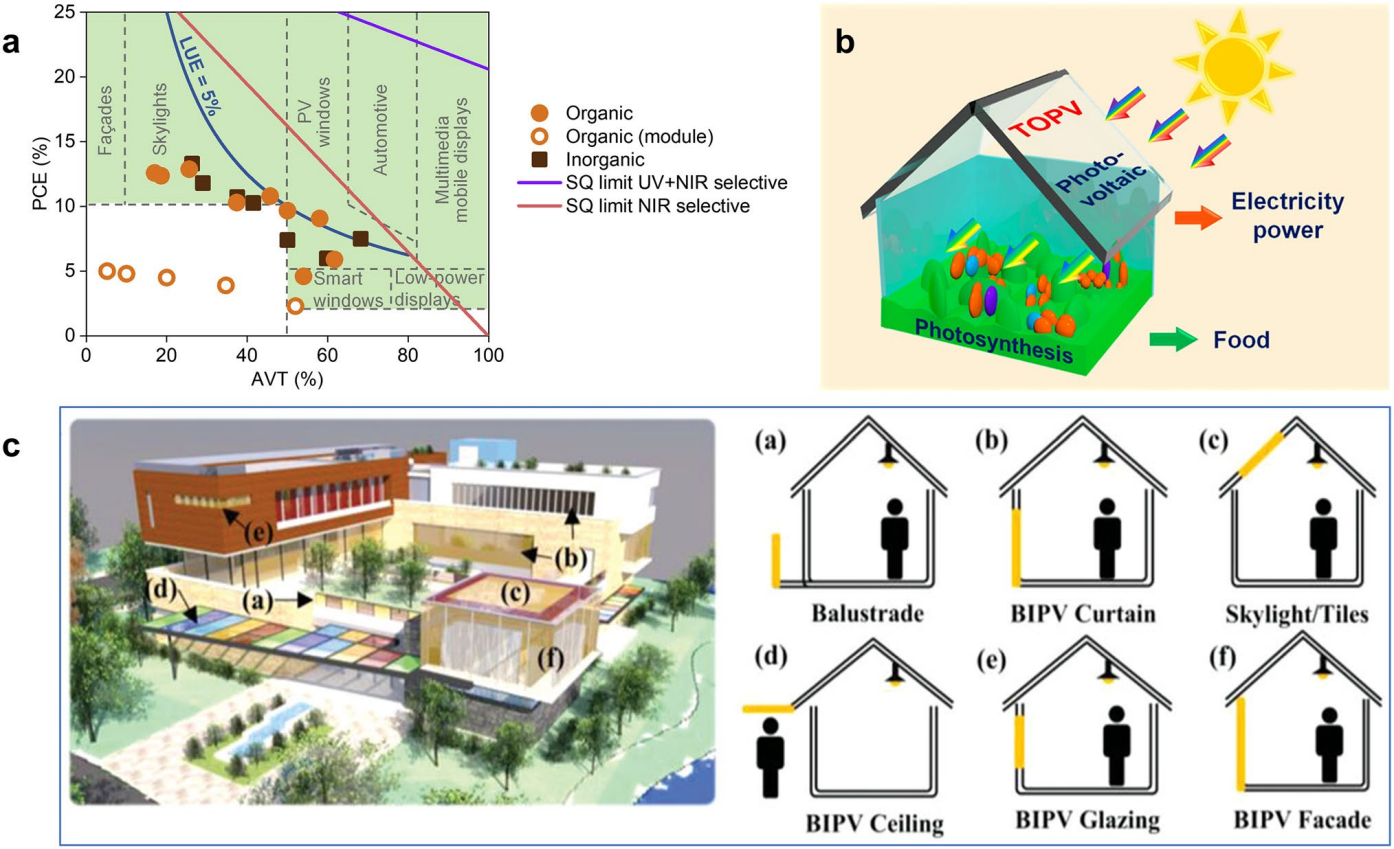
**a** Efficiency and transparency requirements for TPVs in various applications. Reproduced from Ref. [76]. **b** Schematic of TOPV integrated on the roof of greenhouses. Reproduced from Ref. [180]. **c** Building integrated photovoltaics installed at different positions of houses. Reproduced from Ref. [88]

TPVs can be integrated into various applications ranging from small devices such as artificial skin [170, 171] and self-powered sensors [172, 173] to buildings and electrical cars. Those applications have different requirements on transparency and energy demand varying in multiple magnitudes as shown in a TPV performance map for the various applications in Fig. 11a [76]. In the past few years, with the development of the photoactive materials and light management, the state-of-the-art TPVs have reached the requirements of some applications [89, 93, 130, 144]. However, BIPVs remain the primary focus, drawing the most attention in both academia and industry. Therefore, we will focus on various BIPVs for the specific applications of TPVs in this part, such as greenhouse, smart windows, and facade as shown in Fig. 11b, c [87, 88, 174–180]. In addition, we will briefly discuss small scale TPVs for the artificial skin and self-powered sensors for the prospective applications towards electronics.

### Application to BIPVs

As introduced previously, integrating TPVs into building envelopes can relieve the energy crisis in cities by efficiently harvesting solar energy within a crowded space. One facile way to install BIPV is to directly attach to the building envelopes. Flexible TPVs which fit complex curved surface of modern architecture demonstrate a great advantage over rigid panels in this scenario [181]. In addition, flexible TPVs generally have high mechanical stability which is beneficial while faced with environmental loads, such as wind, snow, thermal expansion. However, as TPV technology is still at infancy stage, there lack studies on flexible TPVs and their mechanical properties. Therefore, the following discussion of BIPV focuses on their aesthetic value and potential in energy generation. For a clear presentation, we categorize the BIPVs into greenhouse-integrated photovoltaics, also known as agrivoltaics, smart windows, and facades according to their different requirements. Greenhouse-integrated photovoltaics have special requirements on the spectra of transmittance light, as cultivars need lights of different wavelengths for photosynthesis, development, and growth [176]. The design of agrivoltaics hence favors the wavelength-selective TPVs, such as the LSCs and organic TPVs. While in the application of smart windows and facade, other design criteria such as heat insulation, aesthetics, and privacy protection have a higher priority.

#### Greenhouse-Integrated Photovoltaics

Modern agriculture applies greenhouses to produce various vegetables and crops in all seasons. On one hand, it consumes energy to detect, record, and maintain the temperature and humidity within the greenhouses. On the other hand, many cultivars do not require full sunlight for photosynthesis and growth [182, 183]. Therefore, integrating TPVs into the greenhouses provides a viable method to reduce energy consumption while growing crops.

**Fig. 12:**
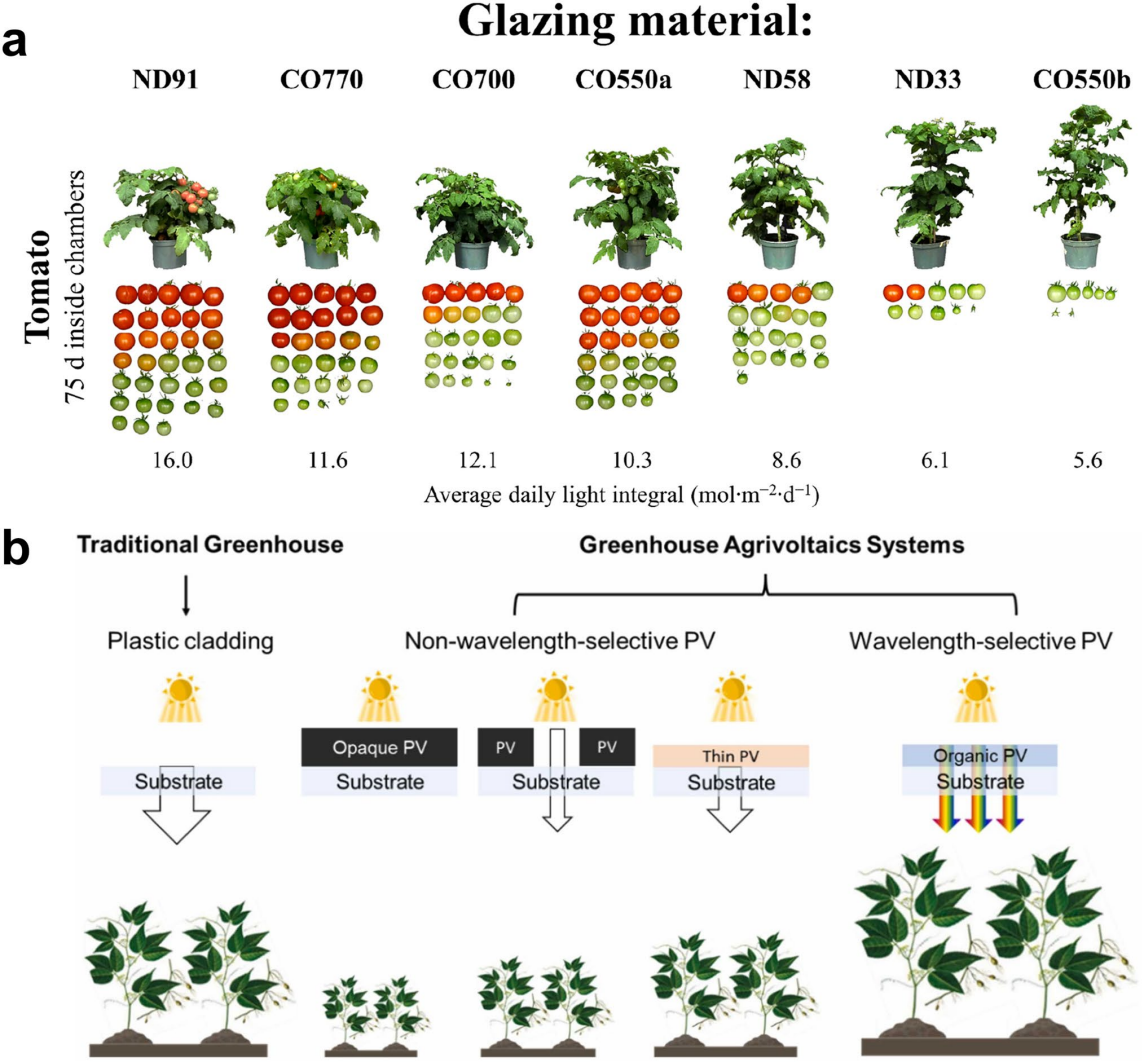
**a** Effect of the amount of daylight passing through different glazing materials on the growth of basil, petunia and tomatoes. Reproduced from Ref. [178]. **b** A scheme showing the different effects between traditional greenhouse, non-wavelength selective PV, and wavelength-selective PV on plant growth. Reproduced from Ref. [184]

Light in the range of 400–700 nm wavelengths is known as photosynthetically active radiation (PAR) which is further divided into blue (400–500 nm), green (500–600 nm), and red (600–700 nm) wavebands [178]. Figure 12a demonstrates the effect of daylight passing through different glazing materials on the growth of three common vegetables, basil, petunia, and tomatoes. In addition, light in other spectra regions, such as UV and NIR, can also play an important role in regulating the growth. For instance, the ratio of the red to NIR region could initiate the shade avoidance response. Plant-specific spectral design is required to optimize power generation and yield of various plant species. Therefore, the wavelength-selective TPVs are more suitable than non-wavelength-selective ones for the agrivoltaics (Fig. 12b) [184].

The organic TPV is a promising candidate for the agrivoltaics because organic materials possess discrete absorption spectra, which has been considered as a drawback in solar farm applications [185]. However, the discrete absorption spectra provide a unique opportunity for achieving both high efficiency and transparency for the agrivoltaics. In addition, the absorption band can be effectively adjusted through the substitutions of various functional groups, internal charge transfer, and extended conjugation length, allowing for crop-specific designs [176, 178, 186]. For instance, Jinnai et al. designed naphthobisthiadiazole-based acceptors (SNTz-RD and ONTz-RD), which mainly absorbed green light while blue and red light passed through the TPVs to promote photosynthesis in strawberry leaves [186].

However, the main issue hindering the application of organic TPVs in the agrivoltaics is their poor operating stability due to the superoxide radicals generated by a metal oxide charge transport layer. Zhao et al. tackled the problem by inserting reduced L-glutathione as an interlayer in the organic TPVs [177]. As a result, devices with the interlayer showed an increase in PCE from 11.6% to 13.5% and maintained over 84% of their initial PCE after continuous illumination for over 1000 h. Meanwhile, the authors also found that mung bean, wheat and broccoli grew in the greenhouses integrated with the organic TPVs had a higher survival rate due to less exposure to the UV light, demonstrating great benefits of the agrivoltaics.

#### Smart Windows and Facades

TPVs are also integrated into windows and facades to generate electricity and modulate indoor light and heat transmission so that a comfortable living environment can be achieved. This particularly becomes an important consideration in the future due to the aggravated extreme weather conditions in recent years. Salameh and coworkers provided a detailed analysis on the cooling load of the BIPV facades [8]. The authors used a PVSYST software to simulate and compare the annual electrical consumption of two commercial buildings with and without BIPVs in Sharjah. After the optimization of various conditions such as building orientation, solar radiation, weather, and wind cooling, the authors achieved 27.69% of energy saving in the building with the BIPV facades.

However, we should also be aware of the complexity involved in designing the BIPVs. On one hand, requirements on PCE and AVT vary with application. Although both smart windows and facades require a PCE over 10% for practical use, facades can be colorful with low transparency while smart windows should have an AVT over 60% with a neutral color to provide a clear view. On the other hand, the performance of the TPVs is influenced by a number of factors, such as light intensity, incident angle, diffusivity, and temperature. Attributed to the large absorption coefficient and high defect tolerance of PVKs, the PVK TPVs show a small *V*_*oc*_ drop under the low light intensity and a weak dependence on the incident angle and diffusivity [5]. These benefits make PVK TPVs attractive in the solar window application. Matteocci and coworkers applied a wide bandgap PVK MAPb(Br_(1−x)_Cl_x_)_3_ and optimized an optical management to achieve a PCE greater than 7% and an AVT greater than 50% in a module with a size of 10.3 cm^2^ [87]. However, the CRI was only about 71.2 as light wavelength below 500 nm was absorbed as well. Ritzer et al. used a laser-scribed micropatterning method to achieve a high CRI of 97 in the PVK TPVs [175]. The developed method could provide multiple levels of transparency and be transferred to more complex tandem structures.

**Fig. 13:**
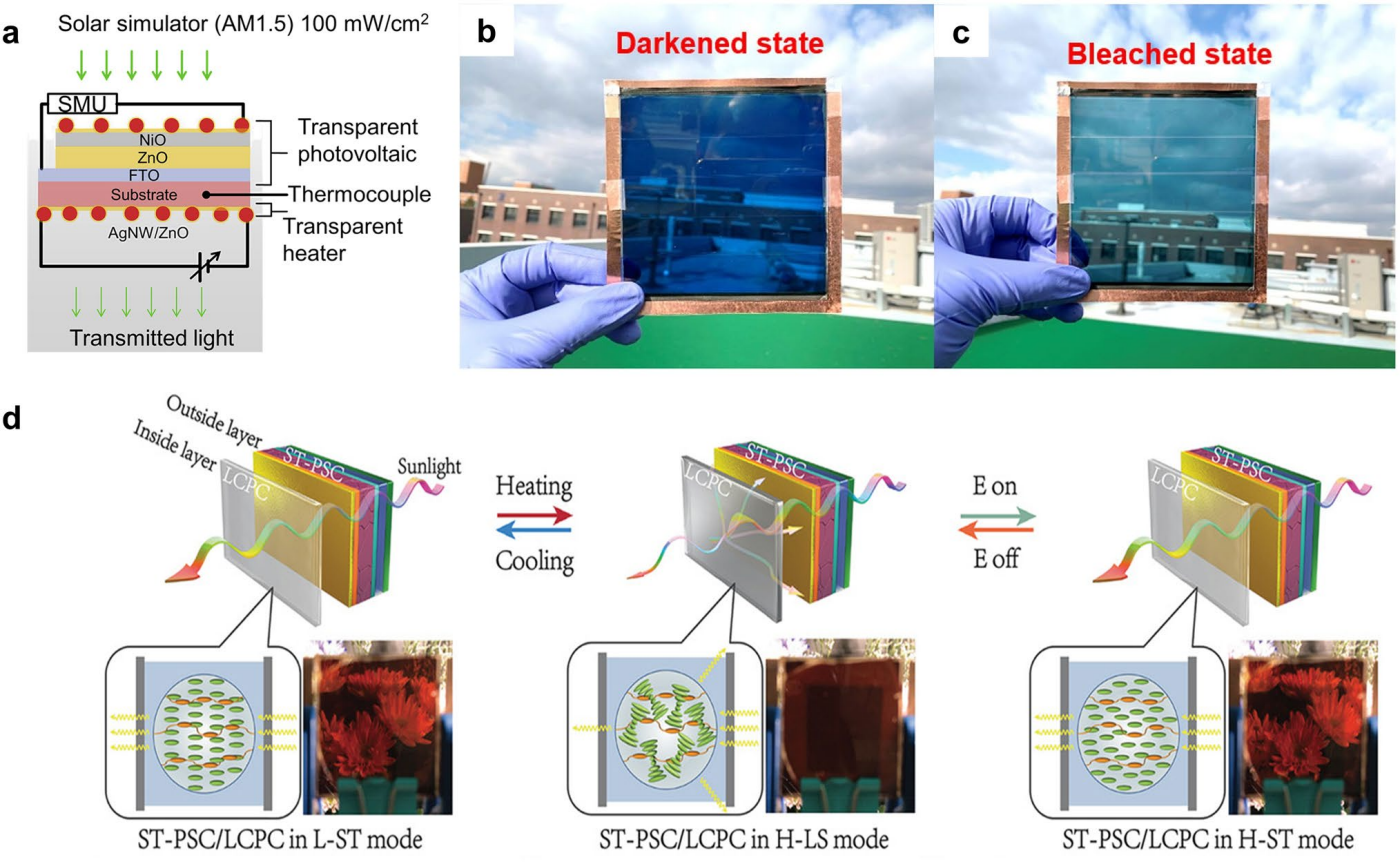
**a** Device structure of NiO/ZnO TPV integrated with Ag nanowire/ZnO transparent heater Ref. [187]. **b, c** Photos showing the integrated OPV/EC module at **b** active (darkened state), and **c** inactive (bleached state). Reproduced from Ref. [188]. **d** Temperature and electric bias changes phase of liquid crystal and therefore transparency of the film. Reproduced from Ref. [189]

While the transparency and electricity generation are priorities for the BIPVs, the smart windows and facades also have other requirements, such as anti-fogging, de-icing, and privacy protection, which the TPVs could hardly realize alone. Therefore, several groups have integrated TPVs with other transparent devices to extend their applications. Patel and coworkers integrated NiO/ZnO TPVs with Ag nanowire/ZnO transparent heaters to realize UV blocking, daylighting, and active heating at the same time as shown in Fig. 13a [187]. The integration of the TPVs and the transparent heaters induced coupling between various thermal processes including joule heating, thermalization, and the Peltier effect, which enhances the de-icing functionality.

Another important issue in the smart windows is privacy protection, which requires switchable on-demand shading. Jeong et al. integrated a large-area organic TPV (100 mm × 100 mm) with a polymer electrochromic (EC) device to realize an active switch between a darkened and bleached state as shown in Fig. 13b, c [188]. Xia and coworkers coupled liquid crystal/polymer composite (LCPC) films with PVK TPVs for privacy protection [189]. As the transparency of the LCPC films was determined by both temperature and electric bias as shown in Fig. 13d, the smart window was fabricated with multi-response and adjustable transparency in the temperature range of 37.8–67 °C. However, the smart window did not have active control on the transparency in the temperature range of − 20–37.8 °C. This drawback may be overcome by employing other liquid crystals or further integrating the device with a transparent heater.

### Other Applications

TPVs also have other interesting applications in scenarios such as bioelectronics and self-powered photodetectors. Bioelectronics, for example, an artificial skin, can be used for continuous real-time health monitoring or provides haptic perceptions for being used in human–machine interfaces. These functions require a sustainable power supply to guarantee portability and long-term operation and thus the TPVs have attracted a significant attention for bioelectronic applications. Núñez and coworkers combined single layer graphene with the TPVs to form an energy-autonomous capacitive pressure sensor [171]. Attributed to the ultralow power consumption of the sensor, energy autonomy was achieved with a PCE of about 1.5% in the work. Bhatnagar et al. studied a NiO/ZnO TPV as an artificial thermoreceptor [170]. They found that the artificial thermoreceptor could detect a wider temperature range than a natural skin due to a pyroelectric current from the ZnO layer. In addition, the thermoreceptor exhibited a hysteresis loop at a high temperature, which can be used as a sensory memory. However, the relatively low PCE due to special requirements for bioelectronic applications needs to be solved in the future.

For the self-powered photodetector application of the TPVs, Nguyen and coworkers reported a high-performance UV detector by employing the pyroelectric effect in ZnO/Cu_2_O TPVs [152]. Cu_2_O possessed a low thermal conductivity and therefore confined photo-induced heat in the ZnO layer. As a result, the self-powered UV detector exhibited a high responsivity of 0.98 A W^−1^, a detectivity of 1.62 × 10^13^ Jones and a microsecond response speed at zero bias.

Recently, TPVs were also applied as transparent neuromorphic devices in image sensing applications. However, the main purpose of the TPVs was shifted from energy generation to offering photo-response with synaptic plasticity. For the completeness of our discussion, below we mentioned some representative work to briefly discuss recent progress in transparent neuromorphic devices. Bhatnagar et al. demonstrated a photo-response increased with the number of successive light pulse in a TPV even with a simple ZnO/NiO heterostructure [190]. The increased photo-response was regarded as a learning process and termed as synaptic plasticity. Similarly, Kumar and coworkers fabricated a TPV with a structure of FTO/TiO_2_/NiO/AgNW and found synaptic plasticity as well [191]. The mechanism of synaptic plasticity was explained by oxygen vacancy migration under electrical bias which modulated the junction width. However, long-range ion migration may cause irreversible damage or even break down devices. To address this challenge, Wu and coworkers applied polyzwitterions in transistor synapses to inhibit long-range ion migration while allowing interfacial charge trapping to provide photo-response [192].

## Perspectives and Conclusion

For a brief summary of the review, the last decade has seen huge progress in the third generation opaque PVs, which also facilitates the development of thin-film TPVs. We introduced wavelength- and non-wavelength-selective strategies to achieve transparency in PVs and discussed the trade-off between the aesthetic requirements and power generation. Then theoretical limits and recent progress in different types of the TPVs were presented, with a particular focus on solution-processed thin-film TPVs. With the aid of light management, the state-of-the-art thin-film TPVs have achieved the LUE over 5% [130] and CRI approaching 100 [94]. In addition, different from silicon, the emerging light absorbers introduced in this review are solution processable and bandgap tunable, enabling a color and transparency customization and large-scale fabrication at a low cost. Then, the applications of TPVs, with stress on agrivoltaics, smart windows and facades were discussed in detail. The results clearly show that the emerging thin-film TPVs are a promising solution to the dilemma of high energy demand and limited space in urban areas.

However, the emerging thin-film TPVs are still at their infancy stage, with the PCE lagging behind their opaque counterparts and transparency below the application requirements. To further improve the performance of TPVs, below we have identified the following topics deserving further investigation in the three types of emerging TPVs.

First, for the PVK TPVs: (1) Methods to deposit uniform ultra-thin PVK films are urgently needed to address the cracks and defects due to inhomogeneous nucleation when the film thickness decreases. The additive and solvent engineering applied in the opaque PSCs may be referred to, especially for the compositions with high chlorine and bromine contents; (2) As PVK possess continuous band structures, their absorption spectra are typically uneven in the visible region, leading to the low CRI. This requires new light management strategies different from opaque PSCs; (3) Current electron and hole transport layers are designed for narrow bandgap PVKs, leading to the large *V*_*oc*_ loss in the TPV application. New electron and hole transport layers are needed to form a better energy band alignment and reduce the *V*_*oc*_ loss; (4) TPVs integrated in buildings or electronics are more accessible in daily life and hence have a more strict requirement on ecotoxicity. Although the toxicity of lead-based PVK is widely discussed in opaque PSCs [193–195], there has been no reports on this challenge in TPVs.

Second, for the organic TPVs: (1) As organic molecules have discontinuous band structures, it is possible to selectively harvest the UV and NIR light to break the PCE limit of the non-wavelength-selective TPVs at the high AVT. To this end, new molecule design principles are needed to adjust the gap between absorption peaks rather than simply redshifting the spectra by inducing a stronger ICT and extending a conjugation length; (2) The discontinuous band structures of organic molecules also provide a unique opportunity in agrivoltaics. The organic absorbers can be judiciously designed to compensate the light spectra required for certain plants to grow. However, so far there have been few studies on such specialized molecule designs and their effects on the plant growth; (3) Although recently many low bandgap donors and acceptors have been developed, the organics typically exhibit weak light absorption. Consequently, judicious light modulation is required to reflect the NIR light back into the cell so that the photocurrent increases without compromising the transparency.

Third, for the CQD TPVs: (1) Although advancements have been made in enhancing the charge carrier mobilities of CQDs, there is still a need for new surface passivation and ligand strategies. These are essential not only for creating uniform compact thin films but also for further improving charge transport properties, especially in CQDs with larger bandgaps; (2) Hot-injection synthesis of the CQDs with a high quality and specific size requires complex purification and careful control of reaction parameters including temperature, composition, and injection rate etc., which is not favorable for large scale commercial applications. A facile synthesis or autonomous synthesis method is urgently needed.

Except for the topics mentioned above, there are other common challenges worth studying in the emerging thin-film TPVs. One challenge is to balance the trade-off between PCE and AVT. The most straight forward strategy to tackle this challenge is to enlarge the bandgap of light absorber, which is commonly applied in PVK TPVs. However, the device efficiency typically drops rapidly with the increased bandgap. Ideally, selective absorption of NIR and UV light can boost PCE without sacrificing AVT, resulting in great interest in multi-junction device structures and organics with discontinuous band structures. In addition to modifying the absorber layer, light management provides a facile way to balance the trade-off between PCE and AVT. Careful optical designs are required to trap UV and NIR light while transmitting visible light. Another challenge is the deposition of top transparent electrodes. On one hand, PVK, organic molecules, and CQD thin films can be easily damaged in the deposition process, for example, by magnetic sputtering of ITO. On the other hand, other transparent electrodes, such as silver nanowires and ultra-thin metal, have higher sheet resistance. A new deposition method of the top transparent electrode is necessary to further improve the performance of the TPVs. One more challenge is the long-term stability required by the potential applications of the TPVs in our daily life. However, all the three emerging thin-film TPVs are faced with instability due to various environmental stress. PVKs are sensitive to moisture and oxygen. CQDs are also vulnerable to oxygen and water, especially when there are surface defects due to ligand exchange. Organic donors/acceptors are relatively more stable in terms of moisture as well as mechanical stress, and therefore more suitable for BIPV applications. However, they may degrade due to superoxide radical generated under light in charge transport layers such as ZnO and TiO_2_. As the development of solution processable thin-film TPVs is still at an infancy stage, most literature still focuses on PCE and AVT of devices, with little attention on the stability of transparent films. New strategies are urgently required to solve the stability issue for future applications. Although the three emerging TPV materials are compatible with mature large-scale solution processing methods, such as blade coating and slot-die coating, leading to a low cost in device manufacturing, synthesis of organics and CQDs may be expensive and raise the overall cost. In addition to improving synthesis method, extending the life time of TPV modules can be another effective way to increase energy return on investment (EROI). Last but not least, manufacturing TPVs in large modules or panels is also a challenging research direction worth studying. On one hand, it is generally difficult to maintain the quality and uniformity of large-area films. On the other hand, it is also not trivial to pattern a module with a high geometric fill factor and limited damage to the device. To address the former issue, solution and additive engineering are promising strategies to suppress heterogeneous nucleation or aggregation so that large-area deposition becomes more controllable. In terms of patterning technology, Huang et al. recently developed a multilevel peel-off method in organic TPVs [23]. The method resulted in a high geometric fill factor of 95.8% with small damage to the device, demonstrating great potential in PVK and CQD TPVs as well.

**Table 1:** A summary of important PVK-based TPVs in recent years

Material	Bandgap (eV)	AVT^b^ (%)	PCE (%)	LUE (%)	CRI/CIE coordinates	V_oc_ (V)	Strategy	References
MAPb(I_0.96_Br_0.04_)_3_	1.62	26.23	18.27	4.79	–	1.10	Pre-crystallization method	[79]
Cs_0.25_FA_0.75_PbI_2.01_Br_0.99_	1.73	20.70^c^	15.50	3.21	–	1.27	Composition engineering	[97]
CsPbI_2_Br	1.90	31.70	14.01	4.44	–	1.21	Intermediate-Adduct-Assisted Growth	[95]
		40.90	10.36	4.24	–	1.22		
Cs_0.1_FA_0.9_PbI_2_Br	1.73	22.2	14.21	3.13	–	1.20	Additive engineering	[118]
FAPbBr_3_	2.23	35.60	5.71	2.03	(0.45, 0.43)	1.24	Optimization of antisolvent timing	[116]
Cs_0.13_FA_0.87_Pb(I_0.87_Br_0.13_)_3_	1.63	22.10	14.10	3.12	–	1.15	Pre-crystallized heterojunction strategy	[78]
FAPbBr_3_	2.23	52.30	5.80	3.03	–	1.41	Blade coating all the layers	[105]
FAPbBr_3_	2.23	45.00	8.60	3.87	97.0	1.42	Intermediate phase transition and blade coating	[88]
MAPbI_3_	1.56	29.00	6.41	1.86	(0.42, 0.45)	1.07	Thermal evaporation	[112]
CsPbBr_3_	2.30	59.80	5.98	3.58	–	1.38	Thermal evaporation	[102]
CsPbCl_2.5_Br_0.5_	2.85	84.60	1.10	0.93	96.5	1.77	Thermal evaporation and composition engineering	[93]
FAMACsPbIBr^a^	1.63	36.04	13.71	4.94	–	1.17	Composite HTL	[101]
(PEA)_2_(Cs_0.015_MA_0.595_FA_0.39_)_39_Pb_40_(Cl_0.8752_Br_0.1248_)_121_	2.92	61.62	0.69	0.43	–	1.10	Composition engineering	[117]
MAPbCl_3_	3.00	72.00	1.06	0.76	(0.33, 0.33)	1.73	Solvent-assisted two step approach	[119]
MAPb(Br_0.87_Cl_0.13_)_3_	2.47	69.70	6.30	4.39	71.2	7.79^e^	Composition engineering with the aid of theoretical calculation	[87]
Cs_0.05_FA_0.83_ MA_0.12_PbBr_0.33_I_2.67_	1.59	32.50	10.53	3.42	(0.37, 0.34)	0.97	Moth-eye-inspired structure for light management	[120]
Cs_0.05_(FA_0.83_MA_0.17_)_0.95_Pb(I_0.83_Br_0.17_)_3_	1.63	22.68^d^	14.78	3.35	–	0.99	interface engineering	[123]
Cs_0.1_FA_0.9_PbI_2_Br	1.73	24.5^d^	14.11	3.45	–	1.23	Interface engineering	[124]
FAPbBr_3_	2.23	70.7	8.1	5.72	60.4	1.73	Interface engineering and anti-reflecting coating	[125]
FAPbBr_2.43_Cl_0.57_	2.36	68.00	7.50	5.10	–	1.53	Composition engineering	[130]
Cs_0.07_(FA_0.85_MA_0.15_)_0.93_Pb(I_0.85_Br_0.15_)_3_	1.60	10.17^d^	15.40	1.57	–	1.03	DMD transparent electrode	[131]

^a^The PVK layer was fabricated via two step spin-coating, detailed composition not reported

^b^AVT is calculated in the range 380–780 nm by default

^c^Calculated in the range 400–800 nm without considering the photopic response

^d^Calculated in the range 400–800 nm

^e^*V*_*oc*_ of a $10 \times 10{\text{ cm}}^{2}$ module. The *V*_*oc*_ of individual cells is not reported

**Table 2:** A summary of important transparent OPVs in recent years

Device structure	AVT^a^ (%)	PCE (%)	LUE (%)	CRI/CIE coordinates	Strategy	References
(LiF/TeO_2_)_4_/glass/ITO/PEDOT:PSS/PM6:BTP-eC9:L8-BO/PDINN/Ag (12 nm)/(LiF/TeO2)^8^/LiF	46.8	11.44	5.35	85.4	Aperiodic band-pass electrode	[89]
Glass/ITO/ZnO/PFN-Br/PCE10:6TSe –OFIC/MoO_x_/Ag(15 nm)	20.0	10.06	2.01	90.4/(0.244, 0.275)	Incorporation of Selenium heterocycles	[73]
Glass/ITO/PEDOT:PSS/66-PTB:IEICO-4F/PDINO/Ag(10 nm)/MoO_3_(50 nm)	50.8	5.11	2.60	74.1/(0.294, 0.338)	Enhance intramolecular charge transfer	[96]
Glass/ITO/PEDOT:PSS/PP2:BTP-eC9/PFN-Br/Au(1 nm):Ag(12 nm)/MoO_3_(50 nm)	43.0	11.10	4.77	(0.343, 0.361)	Solid additive	[137]
Glass/ITO/PEDOT:PSS/PBDB-TF:Y6:BTTPC/PFN-Br/ Ag(12 nm)	22.4	13.10	2.93	(0.272, 0.282)	Ternary blend; distributed Bragg reflector	[138]
Glass/ITO/PEDOT:PSS/PBDB-TF:Y6:BTTPC/PFN-Br/ Ag(14 nm)/LiF(140 nm)/MoO_3_(110 nm)	23.5	12.30	2.89	(0.292, 0.369)		
Glass/ITO/EDT/ZnO/2-ATP/PM6:Y6:PCBM/MoO_3_(15 nm)/Ag(10 nm)/MoO_3_(25 nm)	36.3	10.37	3.85	(0.27, 0.29)	Ternary blend; self-organized interlayer; oxide-metal-oxide electrode	[142]
Glass/ITO/PEDOT:PSS/PTB7-Th:BDTThIT-4F:IEICO-4F/PDIN/Au(1 nm)/Ag(10 nm)	24.6	9.40	2.31	–	Ternary blend	[139]
Glass/ITO/PEDOT:PSS/J52:IEICO-4F:PC71BM/PFN-Br/Ag(15 nm)	19.9	7.75	1.54	(0.317, 0.283)	Ternary blend	[140]
Glass/ITO/PEDOT:PSS/PBT1-S:PTB7-Th:PC71BM/ZrAcac/ Ag(15 nm)	20.8	9.20	1.91	(0.298, 0.328)	Ternary blend	[141]
Glass/ITO/PEDOT:PSS/PBT1-S:PTB7-Th:PC71BM/ZrAcac/ Ag(10 nm)	33.8	8.10	2.74	–		
Glass/ITO/PEDOT:PSS/PBT1-S:PTB7-Th:PC71BM/ZrAcac/ Ag(5 nm)	44.8	5.00	2.24	–		
Glass/ITO/Li:Bphen/BF-DPB:B4PymPm/BF-DPB:F6-TCNNQ/MoO_3_(20 nm)/ITO(40 nm)/TPBi	81.8	0.70	0.57	97.1/(0.337, 0.354)	Conjugation extension; optical outcoupling architecture	[81]
Glass/ITO/PEDOT: PSS/PBOF:eC9:L8-BO/ PNDIT-F3N/Ag	30.5	10.01	3.05	(0.338, 0.313)	Design a polymer donor with an ultrawide bandgap to selectively absorb UV light	[143]
Glass/ITO/PEDOT:PSS/J71:PTB7-Th:IHIC/PDINO/Au(0.8 nm)/Ag(15 nm)/(MoO_3_/LiF)^3^	21.0	9.37	1.97	97	Ternary blend; optical outcoupling architecture	[94]
Glass/ITO/PEDOT:PSS/PTB7-Th:BZO-4Cl/PDINN/Cu(3 nm)/Ag(8 nm)/MoO_3_(46 nm)	43.1	9.33	4.02	90.7/(0.339, 0.361)	Functional group substitution; antireflection layer	[144]
Glass/ITO/ZnO/PM6:BTP-eC9/MoO_3_/Ag(11 nm)/Sb_2_O_3_(40 nm)	26.4	8.77	2.31	(0.279, 0.300)	Optical coupling layer	[146]
Glass/ITO/ZnO NPs/PEIE/PM6: Y6/MoO_3_/Ag(30 nm)/HATCN(70 nm)/Ag(30 nm)	15.6^b^	13.30	2.07	(0.144, 0.118)	Micro-cavity forming electrode	[91]
Glass/ITO/ZnO NPs/PEIE/PM6: Y6/MoO_3_/Ag(30 nm)/HATCN(95 nm)/Ag(30 nm)	10.4^b^	12.90	1.34	(0.295, 0.532)		
Glass/ITO/ZnO NPs/PEIE/PM6: Y6/MoO_3_/Ag(30 nm)/HATCN(120 nm)/Ag(30 nm)	10.5^b^	12.90	1.35	(0.453, 0.266)		
Glass/ITO/PEDOT:PSS/PM6:Y6/Bis-FIMG/Ag(20 nm)/TeO_2_(48 nm)/Ag(18 nm)	31.0^b^	14.04	4.35	(0.172, 0.151)	Fabry–Perot electrode	[92]
Glass/ITO/PEDOT:PSS/PM6:Y6/Bis-FIMG/Ag(24 nm)/TeO_2_(196 nm)/Ag(24 nm)	21.8^b^	14.60	3.18	(0.209, 0.475)		
Glass/ITO/PEDOT:PSS/PM6:Y6/Bis-FIMG/Ag(26 nm)/TeO_2_(104 nm)/Ag(22 nm)	25.2^b^	14.28	3.60	(0.460, 0.287)		
ARC/glass/ITO/ZnO(40 nm)/NSM(1 nm)/PCE-10:BT-CIC:TT-FIC/MoO_3_ (20 nm)/Cu:Ag (16 nm)/CBP (40 nm)/MgF_2_ (100 nm)/CBP (70 nm)/MgF_2_ (40 nm)	44.2	8.00	3.54	(0.280, 0.335)	Ternary blend; optical outcoupling architecture	[83]
PET/Ag grid/D-PH1000/PEDOT:PSS/PM6:Y6:m-BTPPhC6/PDINN/Ag	39.3	11.48	4.51	(0.269, 0.278)	Multilayer flexible electrode; alloyed acceptor; tune the donor–acceptor ratio	[147]
Glass/ITO/PEDOT/PTB7-Th:IEICO-4F/ZnO/Ag NWs	80.4 70.6 55.3	2.38 4.06 5.39	1.91 2.87 2.98	92.4 86.3 82.2	Control film thickness and donor–acceptor ratio	[80]
Glass/ITO/2PACz/PM6:Y6-BO/PNDIT-F3N/Au(0.1 nm)/Ag(8 nm)	30.0	11.30	3.39	–	Self-organized interlayer	[148]
Glass/ITO/2PACz/PM6:Y6-BO/PNDIT-F3N/Ag(15 nm)	19.2	15.20	2.92	–		
Glass/ITO/PEDOT:PSS/PM6/ICBA:Y6/PDINO/Al:Ag(20 nm)	20.4	14.62	2.99	(0.255, 0.280)	Pseudo-planar heterojunction	[106]
Glass/ITO/PEDOT:PSS/D18/N3/PDIN/Au(1 nm)/Ag(10 nm)	22.8	12.58	2.87	–	Layer-by-layer deposition; wide bandgap polymer donor and narrow bandgap NFA	[134]
Glass/ITO/ZnO/PTB7-Th/IEICO-4F/MoO_3_/Ag(12 nm)/Alq3(40 nm)	40.9	8.80	3.60	63.1	Layer-by-layer deposition	[149]

^a^AVT is calculated in the range 380–780 nm by default

^b^The value is the maximum transmittance in the visible range rather than AVT

**Table 3:** A summary of important CQD TPVs in recent years

Material	Ligand	Bandgap (eV)	AVT^a^ (%)	PCE (%)	LUE (%)	CIE coordinates	Strategy	Referencess
PbS	1,2-ethanedithol and PbX_2_ (X = I and Br)	1.29	21.4	8.40	1.80	(0.42, 0.39)	Solution phase ligand exchange	[107]
CsPbBr_3_	guanidinium thiocyanate	2.40	–^b^	5.01	–	(0.436, 0.434)	Solid-state ligand exchange	[104]
PbS	3-mercaptopropionic acid	1.30	22.74	3.08	0.70	(0.478, 0.430)	Ultrathin Au electrode	[164]
PbS	3-mercaptopropionic acid	1.30	24.1	5.40	1.30	(0.46, 0.43)	Symmetric MoO_x_/Au/MoO_x_ structure for light management	[166]
CsPbI_3_	Acetic anion and phenylethylammonium cation	1.78	23.4	11.30	2.64	(0.53, 0.43)	Asymmetric MoO_x_/Au/MoO_x_ structure for light management	[90]
PbS	1,2-ethanedithiol	1.30	26.0	7.40	1.92	–	Interface engineering	[167]
CsPbI_3_	Pb(NO_3_)_2_ and FAI	1.81	53.0	4.95	2.62	–	Graphene electrode	[168]

^a^AVT is calculated in the range 380–780 nm by default

^b^The authors provided transmittance spectra but the AVT value was not reported

**Table 4:** A comparison between PVK, organic, and CQD TPVs (single junction)

TPV type	PVK	Organic	CQD
Typical PCE Range (%)	10–15	8–12	5–10
Typical AVT Range (%)	20–40	30–50	20–30
Best LUE (%)	5.72 [125]	5.35 [89]	2.64 [90]
Advantages	High PCE Bandgap tunability in visible region Weak angular dependence of PCE	Light weight Selective absorption Easy deposition of uniform ultrathin films High CRI	Bandgap tunability from NIR to UV region Inherent compatibility of CQDs
Disadvantages	Poor long-term stability Poor CRI Difficulty in deposition of uniform ultrathin films	Low PCE Poor long-term stability AVT limited by donor content and light scattering in BHJ	Low PCE Poor long-term stability Difficulty in synthesis of high-quality CQDs Extra fabrication process such as ligand exchange
